# Evolutionary and demographic processes shaping geographic patterns of genetic diversity in a keystone species, the African forest elephant (*Loxodonta cyclotis*)

**DOI:** 10.1002/ece3.4062

**Published:** 2018-04-19

**Authors:** Yasuko Ishida, Natalie A. Gugala, Nicholas J. Georgiadis, Alfred L. Roca

**Affiliations:** ^1^ Department of Animal Sciences University of Illinois at Urbana‐Champaign Urbana Illinois; ^2^ Puget Sound Institute University of Washington Tacoma Washington; ^3^ Carl R. Woese Institute for Genomic Biology University of Illinois at Urbana‐Champaign Urbana Illinois

**Keywords:** Congolian forest block, conservation, isolation by distance, landscape genetics, microsatellites

## Abstract

The past processes that have shaped geographic patterns of genetic diversity may be difficult to infer from current patterns. However, in species with sex differences in dispersal, differing phylogeographic patterns between mitochondrial (mt) and nuclear (nu) DNA may provide contrasting insights into past events. Forest elephants (*Loxodonta cyclotis*) were impacted by climate and habitat change during the Pleistocene, which likely shaped phylogeographic patterns in mitochondrial (mt) DNA that have persisted due to limited female dispersal. By contrast, the nuclear (nu) DNA phylogeography of forest elephants in Central Africa has not been determined. We therefore examined the population structure of Central African forest elephants by genotyping 94 individuals from six localities at 21 microsatellite loci. Between forest elephants in western and eastern Congolian forests, there was only modest genetic differentiation, a pattern highly discordant with that of mtDNA. Nuclear genetic patterns are consistent with isolation by distance. Alternatively, male‐mediated gene flow may have reduced the previous regional differentiation in Central Africa suggested by mtDNA patterns, which likely reflect forest fragmentation during the Pleistocene. In species like elephants, male‐mediated gene flow erases the nuclear genetic signatures of past climate and habitat changes, but these continue to persist as patterns in mtDNA because females do not disperse. Conservation implications of these results are discussed.

## 1 INTRODUCTION

Morphological and genetic studies have strongly supported recognition of two African elephant species: the African savanna elephant (*Loxodonta africana*) and African forest elephant (*L. cyclotis*) (Comstock et al., 2002; Groves & Grubb, 2000; Ishida et al., 2011; Roca, Georgiadis, Pecon‐Slattery, & O'Brien, 2001; Rohland et al., 2010). While many studies have indicated that the forest elephant is a species distinct from the savanna elephant, the analysis of genetic diversity below the species level has been limited. Mitochondrial DNA (mtDNA) patterns have been examined in African forest elephants across their range (Debruyne, 2005; Debruyne, Van Holt, Barriel, & Tassy, 2003; Eggert, Rasner, & Woodruff, 2002; Ishida, Georgiadis, Hondo, & Roca, 2013; Johnson et al., 2007; Nyakaana, Arctander, & Siegismund, 2002). Five distinct mitochondrial subclades have been detected among forest elephants, each of which has a different geographically restricted distribution (Ishida et al., 2013). However, several factors can lead to discordant patterns in the phylogeography of nuclear and mitochondrial DNA markers, both within and across species (Petit & Excoffier, 2009; Toews & Brelsford, 2012), and in elephants, there is evidence that female philopatry and male‐biased dispersal combine to produce incongruent mitonuclear patterns (Debruyne, 2005; Roca, Georgiadis, & O'Brien, 2005).

Field studies have shown strong evidence that, despite living in a fission–fusion society, female elephants remain with their closest kin after they mature (Archie, Moss, & Alberts, 2011). Genetic analyses have supported female philopatry by demonstrating almost complete uniformity of mtDNA haplotypes within families (Archie, Fitzpatrick, Moss, & Alberts, 2011). By contrast, male elephants upon reaching maturity disperse from their natal herds (Lee, Poole, Njiraini, Sayialel, & Moss, 2011) and enter periods of musth characterized by competitive interactions with other males for reproductive access to females (Poole, Lee, Njiraini, & Moss, 2011). The dispersal of male elephants from their natal social groups thus mediates nuclear gene flow (Ishida et al., 2011; Nyakaana & Arctander, 1999; Roca et al., 2005, 2015). The phylogeographic patterns revealed by analyses of Y‐chromosome sequences is similar to the pattern for other nuclear markers, but different from patterns shown by mtDNA, supporting the role of males in establishing nuclear phylogeographic patterns (Roca, Georgiadis, & O'Brien, 2007; Roca et al., 2005).

Because mitochondrial phylogeographic patterns are often discordant from nuclear patterns in species in which only males disperse (Petit & Excoffier, 2009; Toews & Brelsford, 2012), including elephants (Roca et al., 2005, 2007), there is a strong need to analyze nuclear markers among forest elephants to examine more completely their evolutionary history and population structure. Furthermore, Central Africa, the region in which most forest elephants live, has suffered from the highest levels of elephant poaching of any subregion within the continent (CITES, 2012), and has been the main source for the illegal trade in elephant bushmeat and ivory (Wasser et al., 2015). Forest elephant numbers declined by ca. 62% between 2002 and 2011, to <10% of their estimated historical population size, mainly due to illegal poaching for their tusks (Maisels et al., 2013). There is thus a strong need to examine fine‐scale population substructure within forest elephants using nuclear markers, for proper conservation management of the species.

Here, we use microsatellite markers to examine nuclear genetic structure in the forest elephant. Ninety‐three individuals from five localities in Central Africa and one individual from Sierra Leone were genotyped using 21 microsatellite markers. We examined nuclear genetic markers for geographic differences among forest elephant localities. We discuss the extent to which regional populations may or may not be genetically distinctive, and the implications of these findings for forest elephant conservation. We also specifically examine the degree of discordance between the phylogeographic patterns inferred using microsatellite markers and patterns previously reported for forest elephant mtDNA across the same tropical forest localities within Central Africa.

## 2 MATERIALS AND METHODS

### 2.1 Samples

**Figure 1:**
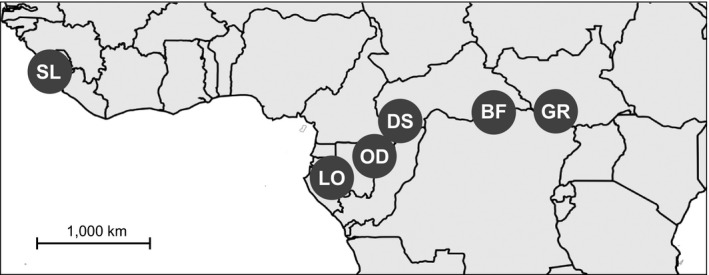
The map shows the sampling locations of forest elephants. Abbreviations are as follows: DS—Dzanga Sangha, Central African Republic; OD—Odzala, Republic of Congo; BF—Bili Forest, Democratic Republic of Congo; LO‐Lope, Gabon; and SL—Sierra Leone (one zoo individual). GR—Garamba in Democratic Republic of Congo is located in the Guinea–Congolian/Sudanian transition zone of vegetation (Olson et al., 2001) that historically included a mixture of forest and secondary grasslands suitable for both African elephant species (Groves & Grubb, 2000)

This study was conducted under the University of Illinois Institutional Animal Care and Use Committee (IACUC)‐approved protocol number 15053. Samples were collected in full compliance with required Convention on International Trade in Endangered Species of Wild Fauna and Flora and other institutional permits. Wild African forest elephants (*L. cyclotis*) were sampled from six localities (Figure 1). Tissue samples were collected primarily by biopsy darting from elephants in Lope (LO) in Gabon, Odzala (OD) in the Republic of Congo, Dzanga Sangha (DS) in the Central African Republic, and Garamba (GR) in the Democratic Republic of Congo. Dung samples of elephants were collected from the Bili Forest (BF) in the Democratic Republic of Congo. A blood sample was obtained from a forest elephant kept at the Paris Zoo that originated in Sierra Leone (SL). These localities represent different geographic regions: Sierra Leone (SL) is located in West Africa; the others are in the Congolian forest block, with LO, OD, and DS to the west, and BF and GR to the east (Figure 1).

In total, 94 forest elephants from six localities (Figure 1) were successfully genotyped at the 21 microsatellite loci (SL: *n *=* *1, LO: *n *=* *15, OD: *n *=* *3, DS: *n *=* *53, BF: *n *=* *3, GR: *n *=* *19) (Tables 1 and S1). As only one sample was available from SL, it was not included in some statistical analyses. Garamba has historically included mixed forest and savanna habitats suitable for both species of African elephant (Groves & Grubb, 2000). Most of our samples from Garamba are forest elephants, although a few are hybrids of savanna and forest elephants based on nuclear genotypes (Comstock et al., 2002; Groves & Grubb, 2000; Ishida et al., 2011; Roca et al., 2001; Rohland et al., 2010).

**Table 1:** Comparison of pairwise *F*_ST_ values calculated using nuclear and mitochondrial DNA

	LO (17)	OD (3)	DS (54)	BF (0)	GR (20)
LO (15)		**0.54**	**0.87**	NA	**0.76**
OD (3)	0.02		**0.49**	NA	**0.38**
DS (53)	**0.02**	0.02		NA	**0.61**
BF (3)	**0.06**	0.06	**0.04**		NA
GR (19)	**0.05**	**0.07**	**0.03**	0.00	

Pairwise *F*_ST_ is shown for comparisons between localities using nuclear microsatellites (below diagonal) and mtDNA (above diagonal). *F*_ST_ values of mtDNA are from Ishida et al. (2013). The values that are significant are indicated in boldface. Sample sizes are in parenthesis for mtDNA (top row) and microsatellites (first column). Localities corresponding to the abbreviations are shown in Figure 1. *F*_ST_ values calculated using mtDNA are not shown for BF as comparable mtDNA sequences are not available for BF.

In addition to the forest elephants, 15 African savanna elephants (*L. africana*) were genotyped, one each from 15 localities (CH—Chobe and MA—Mashatu in Botswana; BE—Benoue and WA—Waza in Cameroon; AB—Aberdares, AM—Amboseli, and KE—Central Kenya/Laikipia in Kenya; NA—Northern Namibia/Etosha; KR—Kruger in South Africa; NG–Ngorongoro, SE–Serengeti, and TA—Tarangire in Tanzania; HW—Hwange, SW—Sengwa, and ZZ—Zambezi in Zimbabwe). Savanna elephant samples were included in light of previous findings that forest and savanna elephants are genetically distinct species with a narrow region of hybridization (Comstock et al., 2002; Groves & Grubb, 2000; Ishida et al., 2011; Roca et al., 2001; Rohland et al., 2010). Because our samples contain forest elephants from Garamba where a few hybrids of savanna and forest elephants have been identified based on nuclear genotypes (Comstock et al., 2002; Ishida et al., 2011; Mondol et al., 2015; Roca et al., 2001), it was determined that the microsatellites would be amplified in savanna elephants, as they would be needed to identify hybrids of savanna and forest elephants. Details on the sampling and DNA extraction have been previously published (Ishida et al., 2011).

### 2.2 Microsatellite genotyping

Allelic variation was examined at 21 microsatellite loci. These markers have been previously developed by Gugala et al. (Gugala, Ishida, Georgiadis, & Roca, 2016). Primer sequences are listed in Table S1. All forward primers included the M13 forward sequence (TGTAAAACGACGGCCAGT) at the 5′ end. The PCR primer mix consisted of a 5′ FAM‐ or VIC‐fluorescent‐labeled M13 forward primer (to label the PCR amplicon), along with the forward primer (with M13 forward sequence at the 5′ end) and reverse primers. The PCR mix included 1× PCR buffer II (Life Technologies, Carlsbad, CA, USA), 2 mmol/l MgCl_2_, 200 μmol/l of each dNTP (Life Technologies) with 0.04 units/μl final concentration of AmpliTaq Gold DNA Polymerase (Life Technologies) along with 1.2 μl of the primer mix. For the DNA samples from BF that had been extracted from dung, 1 μg/μl final concentration of bovine serum albumin (New England BioLabs Inc.) was also included. The PCR cycling program consisted of an initial 95°C for 10 min; with cycles of 15 s denaturing at 95°C, followed by 30 s annealing at 60, 58, 56, 54, or 52°C (two cycles each temperature); or 50°C (last 30 cycles), followed by 45 s extension at 72°C; with a final extension of 30 min at 72°C. For locus Lcy‐M45, the PCR cycling program was modified as described previously (Gugala et al., 2016). PCR amplicons were visualized on a 1.5%–2% agarose gel with ethidium bromide under ultraviolet light. Amplicons of two different loci labeled with different fluorescent dyes (FAM and VIC) were diluted and mixed depending on the intensity of the signal on the agarose gel photograph. Fragment analysis was conducted on the ABI 3730XL capillary sequencer at the University of Illinois at Urbana‐Champaign High‐Throughput Sequencing and Genotyping Unit. The software Genemapper version 3.7 (Life Technologies) was used to call alleles. Relying on a standard of known size, the binning function of the software Genemapper was used to determine fragment lengths, following the procedures indicated in the manual. For the DNA samples extracted from dung (BF), at least four independent amplifications were repeated to confirm homozygotes and three amplifications for heterozygotes (Allentoft et al., 2011).

### 2.3 Characterization of microsatellites

Arlequin version 3.5.1.3 (Excoffier & Lischer, 2010) and GenAlEx 6.5 (Peakall & Smouse, 2012) were used to calculate expected heterozygosity (*H*_*e*_) and observed heterozygosity (*H*_*o*_). The software GenAlEx 6.5 was also used to calculate Shannon's diversity index (*I*) and to make allele frequency distribution histograms for each locus for each locality. Tests for Hardy–Weinberg equilibrium (HWE) and linkage disequilibrium (LD) were conducted on the forest elephant microsatellite data. A Markov chain algorithm was used to test for HWE using 10,000 dememorization steps, 1,000 batches, and 10,000 iterations per batch using the software Genepop 4.2 (Rousset, 2008), and 1,000,000 steps and 100,000 dememorization steps were used with Arlequin version 3.5.1.3. LD was examined using 10,000 dememorization steps, 1,000 batches and 10,000 iterations per batch for each pairwise comparison between loci for Genepop 4.2 and 10,000 permutations for Arlequin version 3.5.1.3.

### 2.4 Population genetic analyses

Analysis of molecular variation (AMOVA) was conducted in Arlequin version 3.5.1.3 (Excoffier & Lischer, 2010) using 10,000 permutations. For each pair of localities, Arlequin was also used to calculate pairwise *F*_ST_ values and statistical support using 10,000 permutations, and to calculate the inbreeding coefficient for each locality, except SL, as only one sample was available from SL.

STRUCTURE 2.3.4 (Pritchard, Stephens, & Donnelly, 2000), which applies a model‐based clustering algorithm to multilocus genotype data, was used to infer population structure using datasets that included or excluded the savanna elephants. STRUCTURE was run eight times for each value of *K* from 1–10, without the use of prior information on locality, under the admixture‐correlated model, with each iteration using at least 1 million Markov chain Monte Carlo generations following a burn‐in of at least 100,000 steps. The uppermost hierarchical level of population structure was examined using an ad hoc statistic *∆K* based on the rate of change in the log probability of the data for a given *K* between successive *K* values, implemented in Structure Harvester (Earl & vonHoldt, 2012). Average coefficients were estimated for each *K* value that was estimated to be uppermost, and for lower values of *K*, employing the Greedy algorithm with 1,000 random input orders as implemented in the program CLUMPP version 1.1.2 (Jakobsson & Rosenberg, 2007). These outputs were visualized using DISTRUCT version 1.1 (Rosenberg, 2004). After identifying the hybrid elephants in GR, we also conducted STRUCTURE analyses excluding them to remove the influence of savanna elephant genotypes. We also conducted STRUCTURE analyses to compare each pair of localities.

Factorial correspondence analyses (FCA) were implemented in GENETIX version 4.05 (Belkhir, Borsa, Chikhi, Raufaste, & Bonhomme, 2004) to graphically plot the distribution of genetic variation for each locality with forest elephants (excluding the hybrids). Principal coordinate analyses (PCoAs) were implemented in GenAlEx 6.5 (Peakall & Smouse, 2012) to visualize the genetic relationships among individual elephants, both including and excluding the savanna elephants and hybrid GR elephants.

### 2.5 Isolation by distance

The coordinate information of each locality was estimated using the LatLong.net website (http://www.latlong.net/), and the pairwise distances between each locality were calculated using the coordinate calculators and distance tools in GPS Visualizer (http://www.gpsvisualizer.com/calculators). To examine the relationship between genetic distances and geographic distances among forest elephants at the five localities in Central Africa, a spatial autocorrelation analysis was implemented in GenAlEx 6.5 (Peakall & Smouse, 2012). The spatial autocorrelation analysis divided the pairwise distances into four ordinal classes and used 9,999 random permutations and 9,999 bootstrap iterations. Isolation‐by‐distance (IBD) analyses were conducted to test the relationships between genetic differences between each pair of localities in Central Africa and the geographic distance between them. These analyses used the Isolation By Distance Web Service Version 3.23 (Jensen, Bohonak, & Kelley, 2005). Two different measures of genetic distance were calculated: *F*_ST_ and Rousset's distance *F*_ST_/(1‐*F*_ST_). Mantel tests were run with 30,000 randomizations (one‐tailed test). For Slatkin's similarity index, we used the recommended log‐transformation of both *M* and geographic distance. As we had only one sample from West Africa (from Sierra Leone, SL), we excluded this sample from analyses.

### 2.6 Phylogenetic analyses

We inferred the phylogenetic relationships among localities using the neighbor–joining (NJ) method implemented in POPTREEW (Takezaki, Nei, & Tamura, 2014). As the number of samples was different among localities, we used *D*_ST_ (Nei, 1972) and *F*_ST_ (Latter, 1972) with sample size bias correction in addition to *D*_A_ (Nei, Tajima, & Tateno, 1983) (for which sample size bias correction was not available) to calculate genetic distances among localities. Support for the nodes in each analysis was assessed using 10,000 bootstrap pseudoreplicates. To exclude the influence of savanna elephant genotypes on GR, forest–savanna hybrid elephants were not included in these analyses. The program FigTree v1.4.2 (Rambaut, 2014) was used to draw trees. To assess the influence of the small sample size of SL (*n *=* *1), we also conducted additional analyses using only one sample from an alternative location (LO). The single LO sample was chosen randomly by RESEARCH RANDOMIZER (https://www.randomizer.org/). Three iterations were run in which a single sample from LO was chosen at random, and the NJ tree was reconstructed with the single LO sample.

## 3 RESULTS

### 3.1 Characterization of forest elephants using microsatellites

Although 21 microsatellite markers were genotyped, three were removed before analyses. Marker Lcy‐M4 had a low genotyping success rate due potentially to null alleles. In marker Lcy‐M15, a 1‐bp indel was detected in some savanna elephants; this marker was removed from the forest elephant analyses as some elephants in Garamba are savanna–forest elephant hybrids. The marker Lcy‐M52 showed a significant deviation from HWE even after Bonferroni correction (*p *<* *.0026), and was monomorphic in three localities. The remaining 18 microsatellite loci (Table S2) did not show deviation from HWE in forest elephants and were used in the population analyses. No significant linkage disequilibrium was detected between pairs of loci after Bonferroni correction.

The mean number of alleles, the mean *H*_*o*_, and the mean *H*_*e*_ of the 18 markers among forest elephants were 7.44 ± 0.79, 0.58 ± 0.05, and 0.61 ± 0.05 respectively. The allele frequencies for each locus are shown in Figure S1 and allele number, heterozygosity, and other information for each marker are listed in Table S2. The markers did not show high diversity in savanna elephants. This would be expected for two reasons. First, the markers had been designed based only on the presence of polymorphisms among forest elephants (Gugala et al., 2016) that are 4–7 million years divergent from savanna elephants (Brandt, Ishida, Georgiadis, & Roca, 2012; Rohland et al., 2010). Additionally, savanna elephants are known to have reduced nuclear genetic diversity relative to forest elephants (Roca et al., 2001; Rohland et al., 2010). In savanna elephants, allele numbers for the 18 microsatellite loci ranged from 1 to 4, with the mean of 2.39 ± 0.24. The mean *H*_*o*_ was 0.21 ± 0.04, and the mean *H*_*e*_ was 0.25 ± 0.05. Allele numbers, heterozygosity, and other information for each marker are listed for the savanna elephants in Table S3.

#### 3.1.1 Analyses of population structure across forest elephant localities

Population genetic analyses involved only forest elephants, except as otherwise indicated. Many analyses were run separately for datasets that included or excluded elephants in GR that were identified as hybrids between forest and savanna elephants, although the outcomes of these analyses were not greatly affected by including or excluding hybrids. Analysis of molecular variance (AMOVA) found that only 3.04% of the variance was accounted for by differences among localities (Table S4). *F*_IT_ was 0.054 (*p *<* *.005) with significant deviation from HWE while *F*_IS_ was 0.024 but did not deviate significantly from HWE. The value calculated for *F*_ST_ was low at 0.030, and this value was statistically significant (*p *<* *.001).

For each pair of forest localities, *F*_ST_ was also calculated (Tables 1 and S5) for pairs of localities (excluding SL for which the sample size was one). We identified five elephants in Garamba as hybrids using STRUCTURE (see below) and removed these five elephants from the analyses. Pairwise *F*_ST_ values were high when each forest locality was compared to savanna elephants, which for these analyses were grouped together (*Laf* in Table S5). In the comparisons involving a forest locality and the grouped savanna elephants, pairwise *F*_ST_ ranged from 0.40 to 0.65, with a statistically significant result for each comparison. *F*_ST_ was also calculated between each pair of localities containing forest elephants (Tables 1 and S5), with values ranging from zero (BF and GR) to 0.07 (GR and OD). *F*_ST_ values were low between pairs of forest elephant localities although there were modest but statistically significant differences between some localities in the eastern and western regions of the Congolian forest block (Tables 1 and S5). Localities with larger sample sizes (DS, GR, and LO) tended to yield statistically significant values. Even when the pairwise differences were found to be statistically significant, the low values for *F*_ST_ suggested that genetic differentiation among forest elephants in the Congolian forest block is modest. When samples from localities in the western forest block were combined and compared to samples combined across the eastern Congolian forest block, the calculated *F*_ST_ was low (0.035), although this value was statistically highly supported (*p *<* *.001). By contrast, *F*_ST_ values that had been previously estimated using only mitochondrial DNA (Ishida et al., 2013) were much higher (Table 1). The *F*_ST_ values calculated using mtDNA ranged from 0.38 to 0.87 for each pair of localities, while pairwise *F*_ST_ determined using microsatellite markers was 0.07 or less.

**Figure 2:**
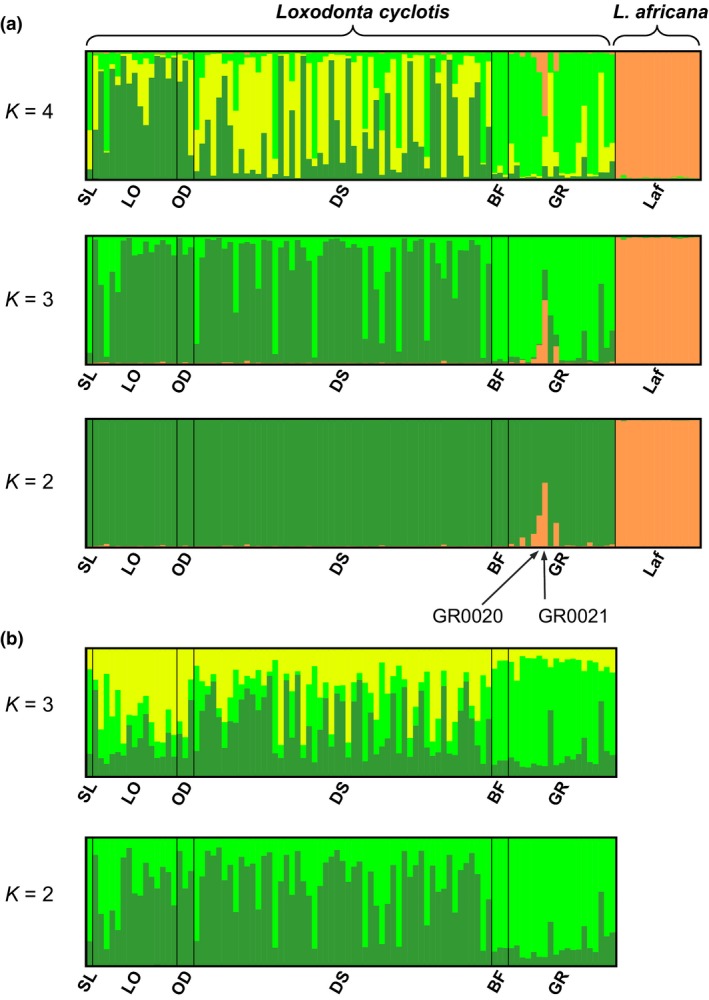
Bayesian clustering approach implemented in STRUCTURE (Pritchard et al., 2000) using 18 microsatellite genotypes, including both forest and savanna elephants or only forest elephants. (a) When both savanna and forest elephants were included, the uppermost *K* value was estimated as four (Earl & vonHoldt, 2012). At *K *=* *2, the forest (*Loxodonta cyclotis*) and savanna (*L. africana*) elephants were almost completely separated into different partitions, with a few hybrids in GR, consistent with previous reports (Ishida et al., 2011). At higher levels of *K*, the additional partitions do not completely separate elephants from different localities, although forest elephants from the eastern Congolian forest (BF, GR) show different patterns in their partitions than localities further west. (b) When only forest elephants were analyzed, the uppermost *K* value was estimated as three. Partitioning within the forest elephants resembles that seen in panel A, with a distinctive but incomplete pattern of partitioning between eastern and western localities. Note that additional analyses (Figure S2) excluding the forest–savanna hybrid elephants from GR did not greatly affect the patterns of partitioning

Analyses using Structure Harvester suggested that the uppermost clustering level was *K *=* *4 when we included savanna elephants and *K *=* *3 when we analyzed using only forest elephants data (Figure 2). The savanna and forest elephants fell into two distinct partitions, with hybrids detected in GR (Figure 2a), consistent with previous reports (Ishida et al., 2011). At higher levels of *K*, the additional partitioning tended to occur between the three localities on the western side of the Congolian forest block (LO, OD, and DS) and the two localities in the eastern side of the Congolian forest block (BF and GR), although partitioning between west and east was incomplete (Figure 2a: *K *=* *3, Figure 2b: *K *=* *2). The STRUCTURE analyses excluding 5 hybrid GR elephants produced similar results (Figure S2). We also conducted STRUCTURE analyses for each pair of localities. We detected differences but incomplete partitioning between GR and the two western localities, DS and LO in the pairwise comparisons (Figure S3). Different patterns of partitioning were not observed between BF and other localities in these pairwise comparisons, presumably due to the small sample size for BF (Figure S3).

A principal coordinates analysis (PCoA) conducted using GenAlEx showed that 25.03% of the genetic variance was explained by coordinate 1, which revealed clear separation between savanna and forest elephant (Figure S5a), except for a GR elephant (GR0021) that was also identified as a forest–savanna elephant hybrid using STRUCTURE (Figure 2a). Factorial correspondence analyses (FCA) implemented using GENETIX (Belkhir et al., 2004) also demonstrated distinctiveness between forest and savanna elephants (Figure S4a). Coordinate 1 explained 64.32% of the genetic variance and clearly separated savanna and forest elephants. In this FCA, western Congolian forest elephants separated from eastern Congolian forest elephants along Coordinate 2 (Figure S4a).

**Figure 3:**
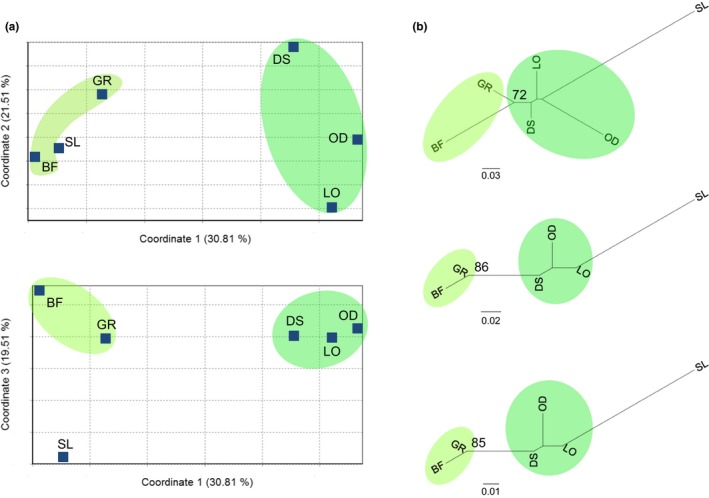
Analyses of forest elephants grouped by locality. Five forest–savanna elephant hybrids from GR were excluded from the analyses. (a) Factorial correspondence analyses implemented in GENETIX version 4.05 (Belkhir et al., 2004) were used to graphically represent the distribution of genetic variation among forest elephant localities. Coordinate 1 explained 30.81% of the genetic variation and separated the Congolian block forest elephants into western (DS, OD, and LO; indicated using darker green) and eastern (BF and GR; indicated using light green) groups. Coordinate 3 explained 19.51% of the genetic variance and separated a single West African Guinean forest block elephant originating in Sierra Leone (SL) from the Central African Congolian forest block elephants. (b) Neighbor–joining trees based on *D*_A_ (top), sample size bias‐corrected *D*_ST_ (middle), and sample size bias‐corrected *F*_ST_ (bottom) showed consistent topologies. Western Congolian forest block localities (DS, OD, and LO, highlighted in darker green) and eastern Congolian forest block localities (BF and GR, highlighted with light green) grouped separately. Bootstrap values ≥ 70% are shown. On all three trees, a clade consisting of the two localities in the eastern Congolian forest block (BF and GR) showed relatively high bootstrap support. Interestingly, a West African elephant from Sierra Leone was separated from the other localities by a long branch, which proved robust regardless of method used to calculate distance or attempts to account for the limited sample size (see Figure S6). Although the distant placement of the Sierra Leone elephant is intriguing, we caution that no strong conclusion can be drawn from a single individual

In PCoAs using only forest elephant data, distinctiveness among localities was not evident when every individual elephant was plotted (Figure S5b). However, when forest genotypes were combined within each locality, FCA separated localities into two groups (Figures 3a, S4a). Coordinate 1 of the FCA explained 30.81% of the genetic variance, separating forest elephant localities into a western Congolian forest group (DS, OD, and LO) and an eastern Congolian forest group (BF and GR). The single sample from Sierra Leone clustered with BF and GR along coordinate 1, but coordinate 3 separated SL from all other forest elephant localities (Figure 3a). Although coordinate 3 explained only 19.51% of the genetic variance and SL consisted of only one elephant sample, this is an intriguing result given that SL consisted of a single sample from the only one of our localities within the Guinean forest block of West Africa, which is not contiguous with the Congolian forest block of Central Africa.

Neighbor–joining (NJ) analyses of forest elephant genotypes grouped by locality produced consistent topologies using three genetic distance calculations: *D*_A_, bias‐corrected *D*_ST_, and bias‐corrected *F*_ST_. The latter two methods correct for biases caused by sample size differences among localities. The analyses involving *D*_A_ tended to show a longer branch for the localities with small sample sizes (Figures 3b, S4b). This tendency was not evident using bias‐corrected *D*_ST_ and *F*_ST_ (Figures 3b, S4b). In all three trees, savanna elephants (*Laf*) formed a lineage distinct from forest elephants (Figure S4b). Among forest elephants, the eastern Congolian forest localities (DS, OD, and LO) formed a clade that was distinct from a clade formed by the western Congolian forest localities (BF and GR), while SL (in the Guinean forest block) had a long branch separating it from all other forest elephant localities (Figures 3b, S4b). To examine whether the separation of SL on the tree was merely due to it having the smallest sample size of *n *=* *1, we reran the analyses while also limiting the sample size of LO to a single individual (Figure S6). Reducing the sample size of LO to one individual affected the trees based on *D*_A_, with the terminal branch length of LO becoming relatively longer. By contrast, trees based on *D*_ST_ and *F*_ST_ that were corrected for sample size bias consistently showed a relatively long branch for SL but not for LO when a single individual was used for each locality (Figure S6). This was true for three different individuals from LO, randomly chosen and used in separate analyses in which the sample size of LO was limited to one. For the two bias‐corrected methods, the long branch length was consistently present for SL with *n *=* *1, but not for LO with *n *=* *1, which may suggest that the long separation between SL and the other populations may be due to actual genetic differences, and not be a mere artifact of the small sample size.

#### 3.1.2 Evidence for isolation by distance among forest elephants in the Congolian forest block

**Figure 4:**
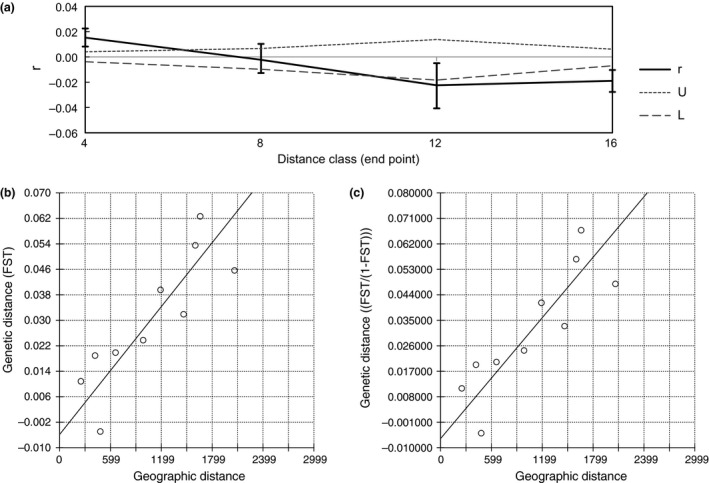
The relationship between genetic distance and geographic distance among forest elephants from different localities. (a) Spatial autocorrelation analysis found that genetic distance was correlated with geographic distance (*r* is the spatial autocorrelation coefficient; *U* is the upper 95% randomization limits of *r*;* L* is the lower 95% randomization limits of *r*). (b) Results consistent with potential isolation by distance were obtained by comparing genetic distance (*F*_ST_) to geographic distance (km) in pairwise comparisons of forest elephant localities (*r *=* *.86, *p *<* *.01). (c) Results consistent with potential isolation by distance were obtained when genetic distances were calculated using Rousset's distance *F*_ST_/(1‐*F*_ST_) and compared to geographic distances (km) in pairwise comparisons of the genotypes of forest elephants grouped by locality (*r *=* *.85, *p *<* *.01). Five forest–savanna elephant hybrids from GR were excluded from the analysis shown in each panel; Sierra Leone (SL) was not used in the pairwise comparisons because the sample size was one. For results including the SL sample, see Figure S7

We examined the degree to which genetic differences among forest elephants at different localities varied with the geographic distances separating them. Geographic distances were computed between each pair of elephants, with the distances placed into quartiles (*x*‐axis in Figure 4a) to implement a spatial autocorrelation analysis (Peakall & Smouse, 2012). Genetic distances between pairs of elephants were also determined (*y*‐axis in Figure 4a). A spatial autocorrelation analysis showed a correlation between genetic distance and geographic distance. Forest elephants that were close to each other geographically were also more similar genetically than were the geographically distant forest elephants (Figure 4a).

Support for isolation by distance (Jensen et al., 2005) was examined. When the single sample from Sierra Leone (SL) was included in the analyses, only a marginally significant correlation between genetic distance and geographic distance was detected for *F*_ST_ (*r *=* *.45, *p *=* *.049, Mantel test) and for Rousset's distance (*r *=* *.45, *p *=* *.052, Mantel test) (Figure S7). However, as SL included only one sample, and SL is from a different and geographically discontinuous forest block, a more conservative approach excluded the single sample from SL for these analyses. Within the Congolian forest block, isolation by distance received strong support, as determined using both *F*_ST_ (*r *=* *.86, *p *<* *.01, Mantel test) (Figure 4b) and Rousset's distance (*r *=* *.85, *p *<* *.01, Mantel test) (Figure 4c).

## 4 DISCUSSION

### 4.1 Current population structure among forest elephants

Nuclear genetic differences were detected among Central African forest locations. Specifically, genetic partitioning by STRUCTURE identified that elephants in western (LO, OD, and DS) and in eastern (BF and GR) Congolian forests were detectably different, although partitioning was far from complete (Figure 2). The same groupings were also evident in clustering analyses (Figure 3). However, as indicated in the PCoA, microsatellite profiles from individuals in eastern and western Central Africa showed a great deal of overlap, and *F*_ST_ values were modest. All of these results point to only limited genetic differentiation between eastern and western localities within the Central African forests.

Hybrids between forest and savanna elephants have been documented, but only within relatively narrow transition zones between forest and savanna habitats (Comstock et al., 2002; Ishida et al., 2011; Mondol et al., 2015; Roca et al., 2001). One important point regards the number of markers needed to estimate the relative contributions of the two species to hybrid individuals. In a previous analysis (Ishida et al., 2011), the hybrid individual shown to have the greatest proportion of savanna elephant alleles was GR0020, whereas the current study identified GR0021 as having the highest savanna elephant contribution (GR0020 had the second highest proportion; Figures 2a and S5a). This likely reflects stochasticity in the genomic distribution of genetic markers from forest or savanna lineages in hybrids, and in the proportion of each genotype attributed to either lineage by the assignment software. The use of a larger number of microsatellite markers, combining the novel markers used here with those previously developed, is likely to provide greater precision in estimating the degree to which hybrids received alleles from one lineage or the other (Boecklen & Howard, 1997). It would also be likely to further increase the accuracy and precision of estimating the provenance of confiscated ivory using nuclear markers (Wasser et al., 2015).

The distinctiveness of elephants from West Africa has been proposed (Eggert et al., 2002), but based largely on mitochondrial DNA data, which can be misleading in elephants (due to maternal inheritance and female philopatry) (Ishida et al., 2011, 2013). The single individual from West Africa (Sierra Leone) in this study appeared to anchor one of the axes in the FCA analysis, and also generated a long branch in the phylogeny, when compared to other isolated individuals (Figures 3 and S4). While suggestive, this is not conclusive evidence for the distinctiveness of West African elephants (a larger sample set is required). However, the Benin/Dahomey Gap separating the Congolian from Guinean forest blocks may hinder gene flow in forest elephants, as it has affected the distribution of other forest‐dwelling taxa (Linder, 2014). For this reason, we previously recommended that a conservative approach would treat elephants on either side of the Gap as deserving of separate conservation status (Roca et al., 2015). Whether Guinean and Congolian forest elephants form genetically distinctive groups (based on nuclear DNA analysis) remains one of the most important unanswered questions in elephant conservation genetics (Roca et al., 2015).

### 4.2 Discordant mitonuclear patterns and the role of range expansion

Mitochondrial DNA patterns have previously been examined in African forest elephants (Debruyne, 2005; Debruyne et al., 2003; Eggert et al., 2002; Ishida et al., 2013; Johnson et al., 2007; Nyakaana et al., 2002), revealing five mitochondrial subclades with distinctive geographically restricted distributions (Ishida et al., 2013). In a previous study (Ishida et al., 2013), pairwise *F*_ST_ values calculated using mtDNA were quite high, ranging from 0.38 to 0.87 when estimated pairwise between localities (Table 1). However, in taxa with male‐biased dispersal, phylogeographic patterns are often discordant between nuclear and mitochondrial DNA (Petit & Excoffier, 2009; Toews & Brelsford, 2012), and discordant mitonuclear patterns have been reported among living and extinct elephantid species (Enk et al., 2011; Lei, Brenneman, Schmitt, & Louis, 2012; Meyer et al., 2017; Palkopoulou et al., 2015; Roca, 2015; Roca et al., 2005). This is consistent with the current analysis, which found nuclear genetic differentiation among forest elephants to be much lower than what might be inferred using mtDNA alone, with all values of *F*_ST_ ≤ 0.07 among forest elephant populations across Central Africa (Table 1). We would note that the high mutation rate of mtDNA would not account for the discrepant patterns, because a faster‐evolving marker would only reveal greater resolution than a slower one; by contrast mtDNA demonstrates a strikingly different phylogeographic pattern than nuclear DNA in forest elephants (Figure 2 vs. Figure S8) as in other elephantids (Enk et al., 2011; Lei et al., 2012; Meyer et al., 2017; Palkopoulou et al., 2015; Roca et al., 2005, 2007, 2015).

Mitonuclear discordant patterns in most cases have been attributed to adaptive introgression of mtDNA, demographic disparities and sex‐biased asymmetries, with some studies also implicating habitat changes and hybrid zone movements (Toews & Brelsford, 2012). In the case of forest elephants, adaptive introgression of mtDNA is unlikely, not only because selective sweeps are unlikely to occur in markers carried only by the nondispersing sex (Petit & Excoffier, 2009), but because mtDNA shows greater differentiation across the forest elephant range than does nuDNA (Table 1). Instead, sex‐based differences in gene flow appear to be responsible for the discordant mitonuclear patterns, with some impact likely due to changes in habitat across geological time. Because female elephants are matrilocal and remain with their natal social group (Archie et al., 2007; Hollister‐Smith et al., 2007), this behavior can account for the persistence of geographic structuring in forest elephant mtDNA (Ishida et al., 2013). By contrast, male elephants disperse from their natal social groups and mediate nuclear gene flow across the landscape (Nyakaana & Arctander, 1999; Roca et al., 2005, 2015).

In forest elephants, the mitonuclear patterns were likely impacted by habitat changes across geological time, and discordant mitonuclear patterns may provide a means for studying their range expansion after the end of the last glacial period. Genetic patterns largely depend on the demographic and ecological characteristics of a species (Castric & Bernatchez, 2003). Spatial patterns of genetic diversity may also reflect past changes in climate and habitats that expanded and contracted the ranges of species, sometimes at a fast pace (Hewitt, 2000). Current spatial genetic diversity may reflect such past events rather than species demography, with geographic differences in genetic diversity reflecting the effects of past climate‐driven range dynamics (Hewitt, 2000). Range expansion can lead to patchiness after migration, due to long‐distance movements followed by population expansions (i.e., a leptokurtic distribution of dispersal distances during colonization) (Ibrahim, Nichols, & Hewitt, 1996; Klopfstein, Currat, & Excoffier, 2006; McInerny, Turner, Wong, Travis, & Benton, 2009). These compounded foundation processes can lead to increased genetic differentiation, although such effects are negatively correlated with migration rate, because migration decreases lags in colonization and reduces the strength of founder effects (Klopfstein et al., 2006; McInerny et al., 2009).

In many species, it is often difficult or impossible to infer processes that are not directly observable from the current spatial genetic structure, especially as various processes may create similar patterns (McIntire & Fajardo, 2009). However, in elephants extreme sex differences in dispersal may allow for the study of both current demographic effects through the examination of male‐mediated nuclear patterns (this study), and for the study of ancient landscape effects through analysis of mitochondrial patterns mediated by females (Figure S8) (Ishida et al., 2013), which may retain signatures of leptokurtic dispersal and compounded foundation processes. Pleistocene glacial cycles caused habitat changes that temporarily isolated populations of some species, with repeated cycles of isolation followed by expansion and contraction (Hewitt, 2000). During periods of spatial expansion, alleles present at the expanding edge of the species range can reach high frequencies (Klopfstein et al., 2006), with expanding populations potentially subject to iterated founder effects (Klopfstein et al., 2006). During expansions, rare long‐distance dispersal events followed by exponential population growth can generate long‐term patchiness in population structure (Hewitt, 2000; Ibrahim et al., 1996). Such ancient events may be preserved in elephant mitochondrial geographic patterns, which are likely to be stable due to female philopatry.

Further, studies of forest elephants would also avoid a common pitfall of using too small a geographic scale (Jenkins et al., 2010) while benefiting from a very large number of museum samples that are available for mtDNA analyses and also have precise provenance information. Species refugia during glacial cycles are better characterized in Europe and North America than elsewhere, and the forest elephant may provide novel insights into the impact of global glacial cycles in the African tropics (Hewitt, 2000).

### 4.3 The potential role of isolation by distance

Distinguishing between discrete population genetic structure (Figures 2 and 3) and isolation by distance (Figure 4) can be difficult (Meirmans, 2012). Models of isolation by distance are often used to approach the balance between drift and dispersal (Jenkins et al., 2010; Wright, 1943). Limited migration permits genetic drift, increasing population differentiation and leading to a correlation between neutral genetic differentiation and geographic distance (Jenkins et al., 2010; Wright, 1943). IBD develops in continuously distributed species when divergence accumulates due to genetic drift between locations separated by geographic distances large enough to overcome the homogenizing effects of gene flow (Chesser, 1983; Crispo & Hendry, 2005; Hewitt, 2000; Jenkins et al., 2010; Wright, 1943), and is common in natural populations (Jenkins et al., 2010). IBD can remain at equilibrium, after sufficient time has elapsed for genetic patterns to be established and stabilized (Castric & Bernatchez, 2003).

Forest elephants have been contiguously distributed across Central Africa from the start of the Holocene (Plana, 2004), until at least the mid‐ to late 1900s (Douglas‐Hamilton, 1987). The correlation between genetic and geographic distances among localities in Central Africa (Figure 4) may be attributable to isolation by distance (IBD) (Jenkins et al., 2010; Wright, 1943). However, because forest elephant populations expanded from discrete glacial refugia, it would be difficult to distinguish a role of IBD from the persistence of discrete population structure (Meirmans, 2012) that would have been diminished but perhaps not erased by postglacial gene flow.

### 4.4 Conservation implications

Given the endangered status of forest elephants, and their role as a keystone species, discussion of the conservation implications of our results is warranted. An important step for the conservation of forest elephants would be universal recognition of its status as a separate species in need of species‐specific conservation measures (Roca et al., 2015). Central Africa, the region in which most forest elephants live, has suffered from the highest levels of elephant poaching of any subregion (CITES, 2012) and has been the main source for the illegal trade in elephant bushmeat and ivory (Wasser et al., 2015) leading to massive declines in their numbers (Maisels et al., 2013). The increase in human numbers and activities has caused fragmentation of elephant habitats and range (Blake et al., 2007, 2008). Such fragmentation reduces genetic connectivity, which can lead to loss of genetic variation, and ultimately to inbreeding and increased drift.

Recognition both of species divisions and of population genetic patterns below the species level is essential for the maintenance of biodiversity, and an important conservation principle is to retain populations representing existing genetic variation (Moritz, 1994; Ryder, 1986). Our findings suggest that West African, western Congolian, and eastern Congolian forest elephants should be managed separately. For species such as the forest elephant that exhibit patterns indicative of limited population structure (Figures 2 and 3) and possibly isolation by distance (Figure 4), Chesser (Chesser, 1983) has suggested dividing the range into management units, with greater genetic exchanges within units than across units in order to balance the need for connectivity with the need to prevent loss of alleles. Within regions, it is important to prevent extreme habitat fragmentation and retain connectivity so that gene flow can continue among populations (Chesser, 1983). Should the destruction of elephants cease while habitats remain, populations could expand from multiple locations. Should active management become necessary for recolonization, the best source populations for translocations would be those that are geographically close, as these would be most similar to any extirpated population (Monsen & Blouin, 2004).

Forest elephants play a critical role in shaping their ecosystem, maintaining tree diversity, dispersing seeds in greater quantities and distances than most other fauna, and improving rates of seed germination following passage through the gut (Campos‐Arceiz & Blake, 2011). In the central Congo Basin, seventy‐eight percent of the larger tree species in the rain forest are dependent on forest elephants for seed dispersal (Blake et al., 2007). Thus, it is important to consider their ecological role in seed germination and dispersal when determining conservation priorities. Terrestrial ecoregions of the world have been mapped to identify areas of high biodiversity and representative communities (Olson et al., 2001). The range encompassed by Central African forest elephants includes five tropical forest ecoregions, three ecoregions of forest–savanna mosaic, as well as the Albertine Rift montane forests and Cameroonian highlands forests (Figure S9) (Olson et al., 2001). Given limited genetic differentiation among elephants across the Congo Basin, these ecoregions could serve as management units for the forest elephants, reflecting the dependence of many plant species on elephants for seed germination and dispersal. Within regions, the extirpation of local elephant populations should be minimized, to minimize effects on the regional flora.

An important further consideration is the impact of forest elephant conservation on those plant species that are rare or regionally restricted. A survey of 5,881 species of plants across sub‐Saharan Africa has been used to map endemism richness, which is defined as the sum of species present at a geographic location, but with the occurrence of each plant species inversely weighted by the size of its range (Figure S10) (Linder, 2014; Linder et al., 2012). The endemic richness of plants has been found to be highly congruent with the endemic richness of frogs, snakes, birds, and mammals, which when combined indicated that the Benin Gap has influenced these patterns (Linder, 2014). Congruence across these groups has been attributed to (1) a similar influence across vertebrates of the vegetation and flora, (2) common responses to the same climatic parameters, and (3) a common underlying history (Linder, 2014). For plants, regions with highest levels of endemism have been identified (Linder, 2014; Linder et al., 2012) and are indicated in Figure S10. These regions of endemism may be considered when setting conservation priorities for forest elephants, although a more precise mapping of endemism among those plants that are dependent on elephants would be helpful. Conserving elephants in the regions rich in plant endemism would directly benefit the conservation of many plant species dependent on elephants, and indirectly benefit other vertebrate and nondependent plant species for which levels of endemism are geographically congruent.

## CONFLICT OF INTEREST

None declared.

## AUTHOR CONTRIBUTIONS

YI and ALR designed the study. YI and NAG performed experiments and analyses. NJG provided samples. YI, NJG, and ALR contributed to writing the manuscript.

## DATA ACCESSIBILITY

The sequences of microsatellites used in this manuscript were established and deposited at NCBI GenBank (KU947083–KU947105), and primer sequences are available in Gugala et al. (Gugala et al., 2016).
