# Supplementary material for: Evolutionary and demographic processes shaping geographic patterns of genetic diversity in a keystone species, the African forest elephant (Loxodonta cyclotis)
Source: Ecol Evol. 2018 Apr 19;8(10):4919–31. doi: 10.1002/ece3.4062 (PMC5980461; doi:10.1002/ece3.4062)
Supplement: Supplementary file 1 [file ECE3-8-4919-s001.pdf]

Evolutionary and demographic processes shaping geographic patterns of genetic diversity in a keystone species, the African forest elephant (*Loxodonta cyclotis*)

Yasuko Ishida[1], Natalie A. Gugala[1], Nicholas J. Georgiadis[2], and Alfred L. Roca[1,3,*]

1 Department of Animal Sciences, University of Illinois at Urbana-Champaign, Urbana, IL, USA
2 Puget Sound Institute, University of Washington, Tacoma WA USA
3 Carl R. Woese Institute for Genomic Biology, University of Illinois at Urbana-Champaign, Urbana, IL, USA

Supplementary information

Table S1. Primer sequences of 21 microsatellites.

Locus	Forward primer sequence (5'-3')	Reverse primer sequence (5'-3')	Allele size range (bp)	Repeat motif	GenBank Accession No.
Lcy-M4	Lcy-M4-F: GGAGAGAGTCTGTGCACCTC	Lcy-M4-R: ATGAGTGTGTGCATGGAACG	122-128	$(AC)_8$	KU947083
Lcy-M6	Lcy-M6-F: ACGGCAATTTAAGATGGAGCC	Lcy-M6-R: CGGTCATTTGCAACACTGTG	130-140	$(AC)_9$	KU947085
Lcy-M8	Lcy-M8-F: TTGAAATTCAGATCAGCGTGTG	Lcy-M8-R: CAGGGCTTTAGTTCACGCTC	125-149	$(TG)_8$	KU947086
Lcy-M15	Lcy-M15-F: AATCAGGCAGCTAACAACGG	Lcy-M15-R: CAGCCCTTCACAGAAAGTCC	148-150	$(TG)_9$	KU947087
Lcy-M16	Lcy-M16-F: CTGATCACTTCTTAGCGGTGTC	Lcy-M16-R: GGTGTTCCTGGACATCTGCC	133-155	$(AC)_{14}$	KU947088
Lcy-M17	Lcy-M17-F: GGAGCTCAGGAAATACACAGC	Lcy-M17-R: TGATCGCCTCTGTTTCGTTC	146-156	$(TG)_{11}$	KU947089
Lcy-M20	Lcy-M20.2-F: GTCTTCCAAAGCACCCTCTG	Lcy-M20.2-R: AATAGACGGGAGAGGGGAGT	156-158	$(AC)_{10}$	KU947090
Lcy-M22	Lcy-M22-F: CTTGAGCCTGCTGTATGTGC	Lcy-M22-R: CAGAAGCTGGATGGTCAAGC	155-157	$(TG)_{10}$	KU947091
Lcy-M23	Lcy-M23.2-F: TGAAGGCGTCTCTGTTATTGC	Lcy-M23.2-R: GAAGCCAAGCAAATGGGATA	160-168	$(AC)_{12}$	KU947092
Lcy-M24	Lcy-M24.2-F: GATGACTCTGGCTCCTGGAT	Lcy-M24.2-R: CGCCCTGACTGGTCTTACTC	114-132	$(TG)_{16}$	KU947093
Lcy-M26	Lcy-M26-F: CAACCAAGTTCTGCCTGCTG	Lcy-M26-R: CGTGGTCTCTTTGCTGTCAC	148-162	$(AC)_{10}$	KU947094
Lcy-M27	Lcy-M27.2-F: TCCTTGATGTGGTTTCAGTCA	Lcy-M27.2-R: GGTAGGTTGGCTATGTTTCTTG	123-131	$(AC)_9$	KU947095
Lcy-M29	Lcy-M29-F: AGCCTCTTTCTTTCCGTTGC	Lcy-M29-R: AGATCCTCAGGGTAATTTGCAC	160-170	$(TG)_8$	KU947096
Lcy-M30	Lcy-M30-F: CACTACGCCAACAGGTTTCC	Lcy-M30-R: TGTCCCATAGTGTCACCGTG	150-156	$(AC)_8$	KU947097
Lcy-M39	Lcy-M39.2-F: CATGGTGACTGCTGCATTG	Lcy-M39.2-R: TGTTGCTTGTTCTGTCCCTAGA	157-163	$(AGC)_{10}$	KU947098
Lcy-M40	Lcy-M40-F: TTCAAGTACCTGCTGTCACTG	Lcy-M40-R: TTCTGTCCAAGTAGCTTACCTG	160-166	$(AC)_9$	KU947099
Lcy-M43	Lcy-M43.2-F: TCCTCACTGCCACAACCAT	Lcy-M43.2-R: GGGTCTCTTCCCATCATCAG	135-155	$(AC)_{26}$	KU947101
Lcy-M44	Lcy-M44.2-F: GGCCTGATATAGCCCTTGT	Lcy-M44.2-R: TCCTGCTGTACTTGAATTTCTGA	128-144	$(AC)_{11}$	KU947102
Lcy-M45	Lcy-M45.3-F: CAGCTATTCTATCCTCACAAGTCCT	Lcy-M45.3-R: TGTGAATGGAGAGTTTTCTCTGA	123-147	$(AC)_{18}$	KU947103
Lcy-M50	Lcy-M50.2-F: CCCAGGACCAGCATAGTGAT	Lcy-M50.2-R: TGGAATAAATCAAGAGTAAGAATTCAC	137-147	$(AG)_{11}$	KU947104
Lcy-M52	Lcy-M52-F: CCGTAAGCTAATCCTCCTGC	Lcy-M52-R: ATTTGCCTTTGTTCCTGCCG	168	$(AC)_8$	KU947105

An M13 forward tail (TGTAAAACGACGGCCAGT) that was at the 5' end of each forward primer is not shown above.

Allele sizes shown here are base on forest elephants in Lope National Park, Gabon (Gugala et al. 2916) and include both forward and reverse primer lengths including the M13 forward tail length.

Table S2. Characterization of 21 microsatellite loci in each forest elephant locality.

	DS					GR					LO					OD					SL					BF					Total				
Locus	*n*	*Na*	*I*	*Ho*	*He*	*n*	*Na*	*I*	*Ho*	*He*	*n*	*Na*	*I*	*Ho*	*He*	*n*	*Na*	*I*	*Ho*	*He*	*n*	*Na*	*I*	*Ho*	*He*	*n*	*Na*	*I*	*Ho*	*He*	*n*	*Na*	*I*	*Ho*	*He*
M6	53	5	1.41	0.75	0.72	19	5	1.50	0.74	0.76	15	5	1.33	0.80	0.70	3	5	1.56	1.00	0.78	1	1	0.00	0.00	0.00	3	3	0.87	0.67	0.50	94	5	1.45	0.76	0.74
M8	53	6	1.33	0.60	0.69	19	4	0.86	0.47	0.52	15	6	1.42	0.67	0.70	3	3	1.01	0.67	0.61	1	1	0.00	0.00	0.00	3	4	1.24	0.67	0.67	94	8	1.37	0.59	0.69
M16	53	10	1.94	0.85	0.83	19	7	1.52	0.68	0.70	15	5	1.26	0.67	0.66	3	3	0.87	0.33	0.50	1	1	0.00	0.00	0.00	3	2	0.64	0.00	0.44	94	11	1.93	0.73	0.81
M17	53	7	0.99	0.45	0.44	19	6	0.93	0.47	0.43	15	4	0.73	0.47	0.38	3	2	0.45	0.33	0.28	1	1	0.00	0.00	0.00	3	2	0.45	0.33	0.28	94	8	0.97	0.45	0.42
M20	53	2	0.37	0.17	0.22	19	4	0.65	0.37	0.32	15	2	0.54	0.47	0.36	3	1	0.00	0.00	0.00	1	1	0.00	0.00	0.00	3	3	0.87	0.33	0.50	94	4	0.51	0.26	0.27
M22	53	4	0.27	0.11	0.11	19	3	0.88	0.32	0.54	15	2	0.24	0.13	0.12	3	1	0.00	0.00	0.00	1	1	0.00	0.00	0.00	3	2	0.64	0.67	0.44	94	4	0.48	0.17	0.24
M23	53	4	0.95	0.58	0.54	19	7	1.70	0.79	0.79	15	4	0.87	0.60	0.46	3	3	1.01	1.00	0.61	1	2	0.69	1.00	0.50	3	2	0.64	0.67	0.44	94	7	1.25	0.65	0.62
M24	53	10	1.84	0.75	0.78	19	8	1.86	0.84	0.82	15	5	1.43	0.80	0.74	3	3	1.01	0.33	0.61	1	1	0.00	0.00	0.00	3	4	1.33	1.00	0.72	94	10	1.85	0.77	0.80
M26	53	8	1.58	0.68	0.73	19	5	1.13	0.63	0.61	15	7	1.85	0.80	0.83	3	2	0.64	0.67	0.44	1	2	0.69	1.00	0.50	3	2	0.45	0.33	0.28	94	8	1.62	0.68	0.75
M27	53	4	0.77	0.43	0.44	19	2	0.21	0.11	0.10	15	3	0.80	0.53	0.46	3	3	0.87	0.67	0.50	1	2	0.69	1.00	0.50	3	2	0.45	0.33	0.28	94	4	0.72	0.39	0.39
M29	53	4	1.03	0.66	0.57	19	7	1.63	0.89	0.77	15	5	1.22	0.47	0.65	3	3	0.87	0.67	0.50	1	2	0.69	1.00	0.50	3	2	0.45	0.33	0.28	94	8	1.27	0.67	0.65
M30	53	3	0.98	0.55	0.58	19	4	1.15	0.47	0.63	15	3	0.87	0.60	0.50	3	3	1.01	0.33	0.61	1	2	0.69	1.00	0.50	3	3	0.87	0.67	0.50	94	4	1.02	0.54	0.59
M39	53	5	0.71	0.34	0.33	19	6	1.16	0.53	0.54	15	2	0.45	0.07	0.28	3	3	0.87	0.67	0.50	1	1	0.00	0.00	0.00	3	2	0.64	0.67	0.44	94	7	0.89	0.35	0.39
M40	53	4	0.86	0.55	0.53	19	2	0.69	0.58	0.50	15	3	0.88	0.47	0.55	3	2	0.45	0.33	0.28	1	2	0.69	1.00	0.50	3	2	0.64	0.67	0.44	94	4	0.84	0.54	0.52
M43	53	11	2.15	0.81	0.87	19	14	2.42	0.84	0.90	15	8	1.83	0.87	0.81	3	3	1.01	0.33	0.61	1	2	0.69	1.00	0.50	3	4	1.24	0.67	0.67	94	15	2.29	0.81	0.88
M44	53	9	1.86	0.68	0.81	19	8	1.89	0.84	0.82	15	6	1.57	0.80	0.77	3	3	1.01	0.67	0.61	1	2	0.69	1.00	0.50	3	5	1.56	1.00	0.78	94	10	1.94	0.74	0.83
M45	53	10	1.99	0.85	0.84	19	9	1.92	0.89	0.83	15	8	1.86	0.87	0.82	3	3	1.01	0.33	0.61	1	1	0.00	0.00	0.00	3	6	1.79	1.00	0.83	94	13	2.10	0.84	0.85
M50	53	4	0.84	0.57	0.47	19	3	0.67	0.47	0.38	15	4	1.02	0.60	0.56	3	2	0.64	0.00	0.44	1	2	0.69	1.00	0.50	3	1	0.00	0.00	0.00	94	4	0.85	0.52	0.47
M4	52	5	0.83	0.33	0.40	17	3	0.83	0.24	0.51	15	4	0.82	0.27	0.43	3	2	0.69	0.33	0.50	1	2	0.69	1.00	0.50	3	2	0.45	0.33	0.28	91	5	0.91	0.31	0.45
M15	53	4	0.61	0.30	0.31	19	3	0.51	0.32	0.27	15	2	0.15	0.07	0.06	3	2	0.45	0.33	0.28	1	2	0.69	1.00	0.50	3	2	0.45	0.33	0.28	94	4	0.55	0.28	0.27
M52*	53	2	0.05	0.02	0.02	19	4	0.65	0.26	0.32	15	1	0.00	0.00	0.00	3	2	0.64	0.00	0.44	1	1	0.00	0.00	0.00	3	1	0.00	0.00	0.00	94	5	0.28	0.06	0.10
Mean		5.81	1.12	0.53	0.54		5.43	1.18	0.56	0.57		4.24	1.01	0.52	0.52		2.57	0.77	0.43	0.46		1.52	0.36	0.52	0.26		2.67	0.75	0.51	0.43		7.10	1.20	0.53	0.56
SE		0.61	0.13	0.05	0.05		0.62	0.13	0.05	0.05		0.44	0.12	0.06	0.05		0.19	0.08	0.07	0.04		0.11	0.08	0.11	0.06		0.28	0.10	0.07	0.05		0.70	0.13	0.05	0.05

n : sample size, *Na* : allele numbers, *I* : Shannon's information index, *Ho* : observed heterozygosity, *He* : expected heterozygosity.
*Deviation from HWE after Bonferroni correction.

Table S3. Characterization of 21 microsatellite loci in savanna elephants.

Locus	*n*	*Na*	*I*	*Ho*	*He*
M6	15	3	0.53	0.27	0.29
M8	15	2	0.45	0.33	0.28
M16	15	4	0.78	0.33	0.39
M17	15	4	0.94	0.47	0.52
M20	15	2	0.15	0.07	0.06
M22	15	3	0.53	0.33	0.29
M23	15	2	0.15	0.07	0.06
M24	15	4	1.15	0.40	0.61
M26	15	2	0.64	0.00	0.44
M27	15	1	0.00	0.00	0.00
M29	15	1	0.00	0.00	0.00
M30	14	1	0.00	0.00	0.00
M39	15	2	0.33	0.20	0.18
M40	15	1	0.00	0.00	0.00
M43	15	3	0.47	0.27	0.24
M44	15	2	0.69	0.53	0.50
M45	15	3	0.77	0.47	0.49
M50	15	3	0.39	0.13	0.18
M4	15	2	0.39	0.13	0.23
M15	–	–	–	–	–
M52*	15	2	0.39	0.13	0.23
Mean		2.35	0.44	0.21	0.25
SE		0.22	0.07	0.04	0.04

n : sample size, *Na* : allele number, *I* : Shannon's information index, *Ho* : observed heterozygosity, *He* : expected heterozygosity
*Deviation from HWE after Bonferroni correction.

Table S4. Analysis of molecular variation among forest elephants.

Source of variation	*df*	Sum of squares	Variance components	Percentage of variation
Among localities	5	44.84	0.17	3.04
Among individuals within localities	88	453.10	0.13	2.34
Within individuals	89	463.00	5.20	94.63
Total	177	960.938	5.49773	

Hybrid elephants from Garamba were not included in this analysis.

Table S5. Population pairwise $F_{ST,}$ within-population inbreeding coefficient, and the pairwise distances.

	LO	OD	DS	BF	GR
LO (15)	0.01	417.8	659.7	1601	2063
OD (3)	0.02	0.22	250.7	1190	1659
DS (53)	**0.02**	0.02	0.02	985.4	1463
BF (3)	**0.06**	0.06	**0.04**	0.02	480.3
GR (19)	**0.05**	**0.07**	**0.03**	0.00	0.03
Laf (15)	**0.47**	**0.59**	**0.43**	**0.57**	**0.45**

F_{ST} is shown below the diagonal for pairwise comparisons; those values that are significant are indicated in bold. The diagonal indicates the inbreeding coefficient (F_{IS}) at forest elephant localities (underlined). Geographic distances in km are shown above the diagonal. SL was not included since the sample size was one. The sample size is in parentheses; localities in the western Congolian forest block are shaded.

Figure S1. Histograms showing the sizes and frequencies of alleles at each microsatellite locus for each geographic locality. Five hybrids from GR were excluded.

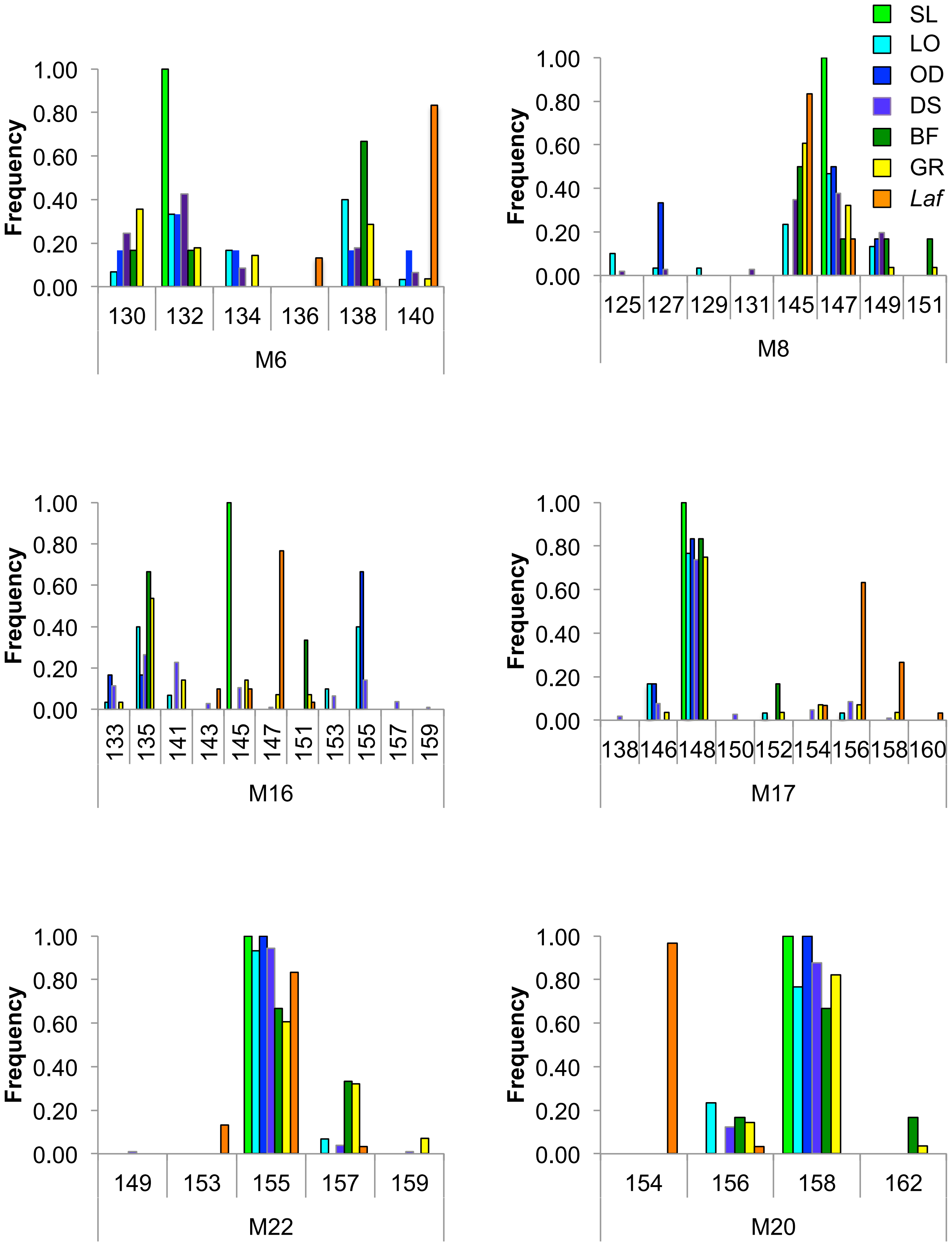
SL
LO
OD
DS
BF
GR
Laf
Frequency
1.00
0.80
0.60
0.40
0.20
0.00
M6
130 132 134 136 138 140
M8
125 127 129 131 145 147 149 151
M16
133 135 141 143 145 147 151 153 155 157 159
M17
138 146 148 150 152 154 156 158 160
M22
149 153 155 157 159
M20
154 156 158 162

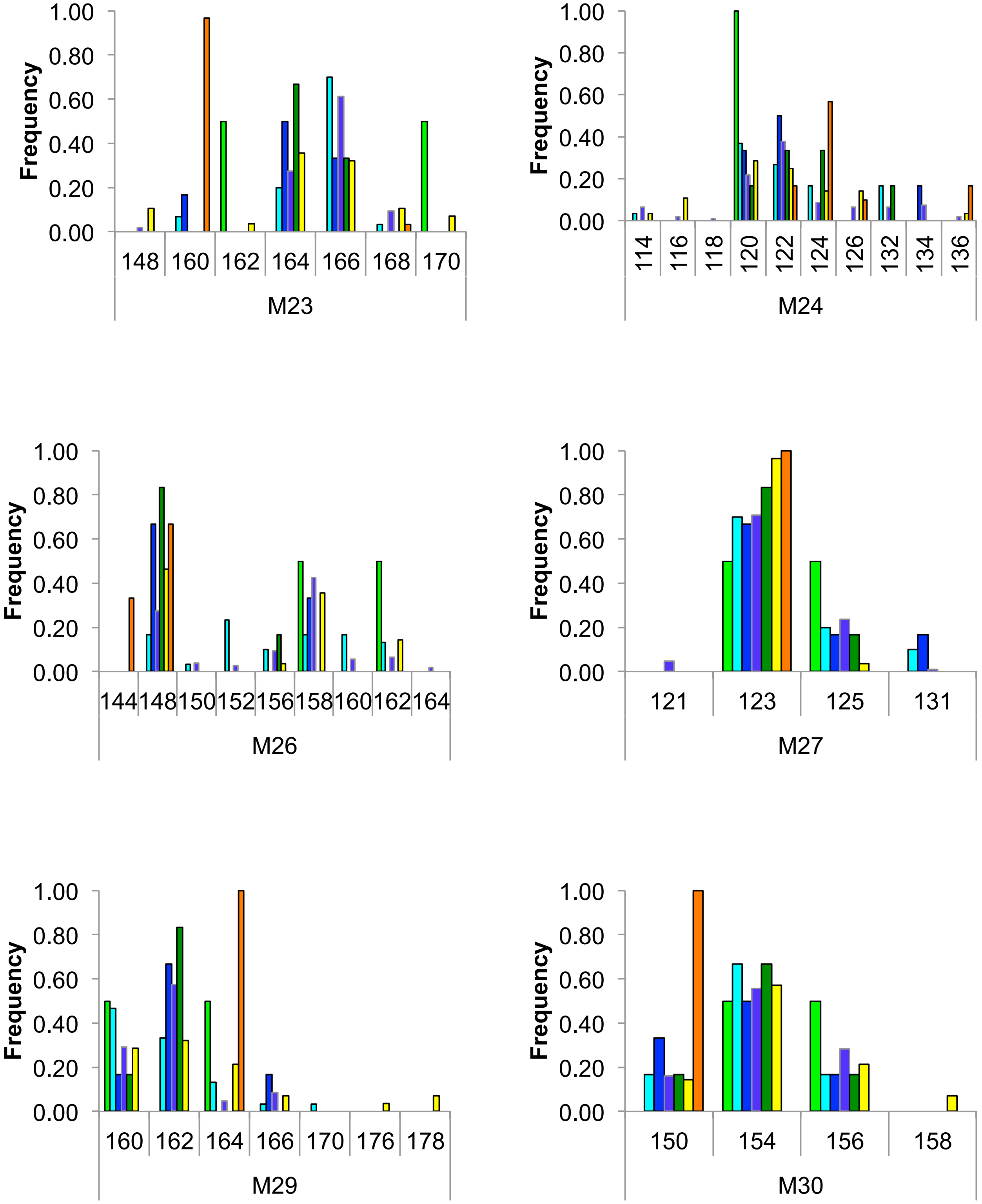
Frequency
1.00
0.80
0.60
0.40
0.20
0.00
148 160 162 164 166 168 170
M23
114 116 118 120 122 124 126 132 134 136
M24
144 148 150 152 156 158 160 162 164
M26
121 123 125 131
M27
160 162 164 166 170 176 178
M29
150 154 156 158
M30

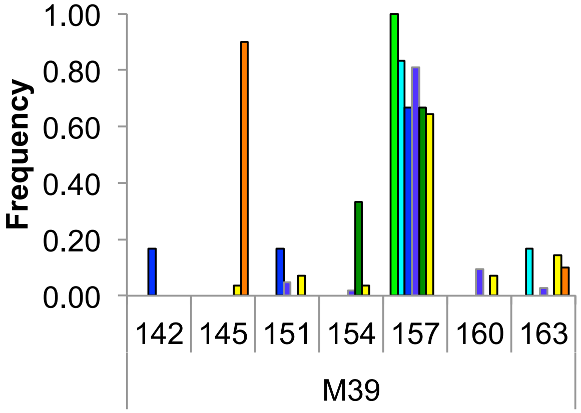
Frequency
1.00
0.80
0.60
0.40
0.20
0.00
142 145 151 154 157 160 163
M39

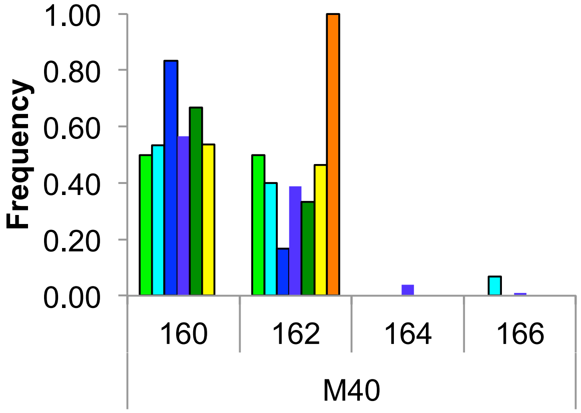
Frequency
1.00
0.80
0.60
0.40
0.20
0.00
160 162 164 166
M40

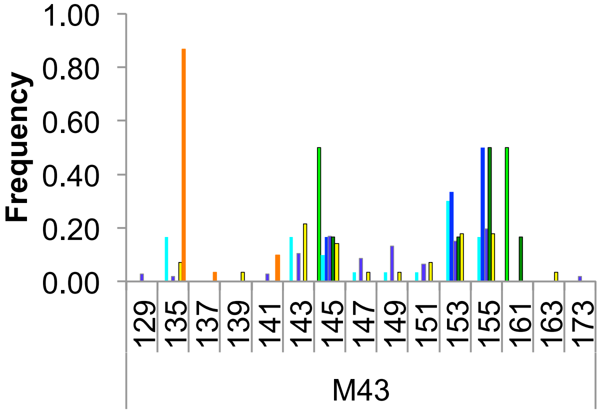
Frequency
1.00
0.80
0.60
0.40
0.20
0.00
129 135 137 139 141 143 145 147 149 151 153 155 161 163 173
M43

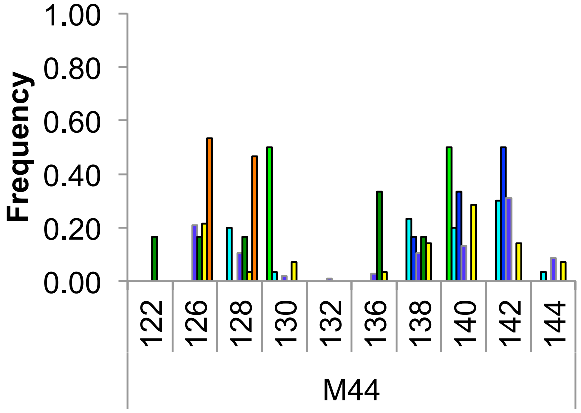
Frequency
1.00
0.80
0.60
0.40
0.20
0.00
122 126 128 130 132 136 138 140 142 144
M44

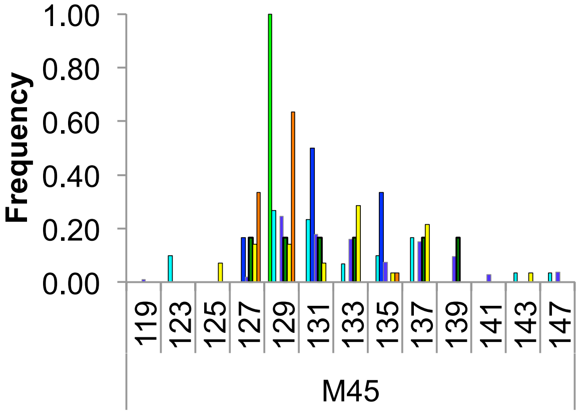
Frequency
1.00
0.80
0.60
0.40
0.20
0.00
119 123 125 127 129 131 133 135 137 139 141 143 147
M45

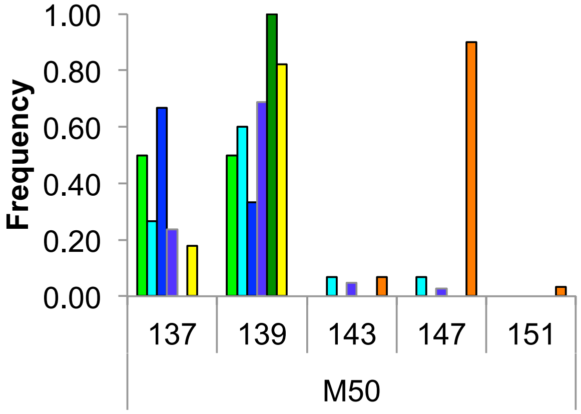
Frequency
1.00
0.80
0.60
0.40
0.20
0.00
137 139 143 147 151
M50

Figure S2. Bayesian clustering approach implemented in STRUCTURE (Pritchard *et al.* 2000) using 18 microsatellite genotypes, using both forest and savanna elephants or only forest elephants. These analyses include the forest-savanna hybrid elephants from GR-Garamba. (A) When both savanna and forest elephants were included, the uppermost *K* value (Earl & vonHoldt 2012) was estimated as four. (B) When only forest elephants were analyzed, the uppermost *K* value was estimated as three. Partitioning within the forest elephants resembles that seen in panel A, with a distinctive but incomplete pattern of partitioning between eastern and western localities.

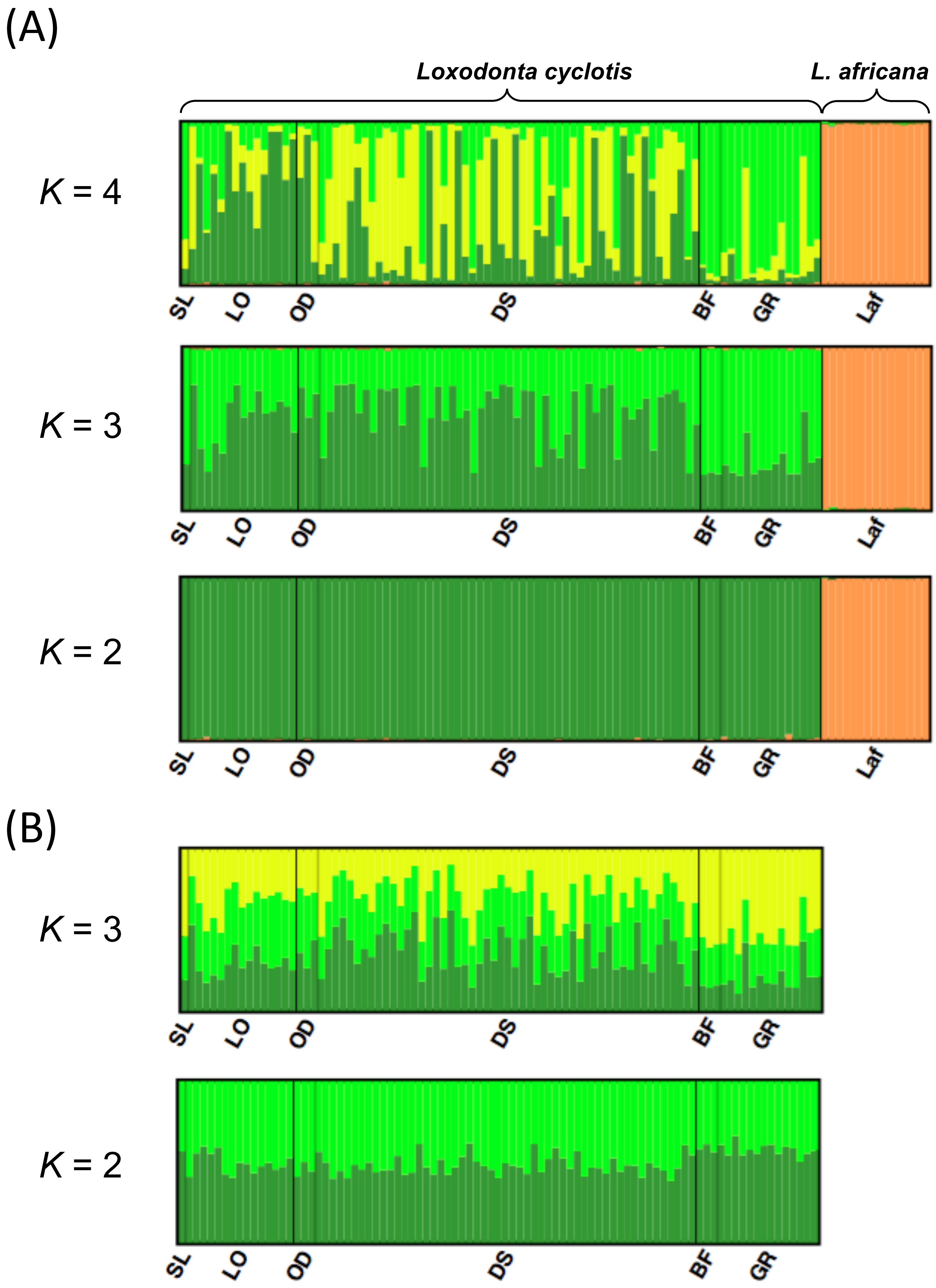

(A)
Loxodonta cyclotis
L. africana
K = 4
K = 3
K = 2
SL
LO
OD
DS
BF
GR
Laf
(B)
K = 3
K = 2

Figure S3. STRUCTURE analyses ($K = 2$) using pairs of localities. Different patterns in the partitions were evident between DS and GR, and between LO and GR. It is possible that a difference between BF and western Congolian forest elephant localities was not observed due to the small sample size of BF ($n = 3$).

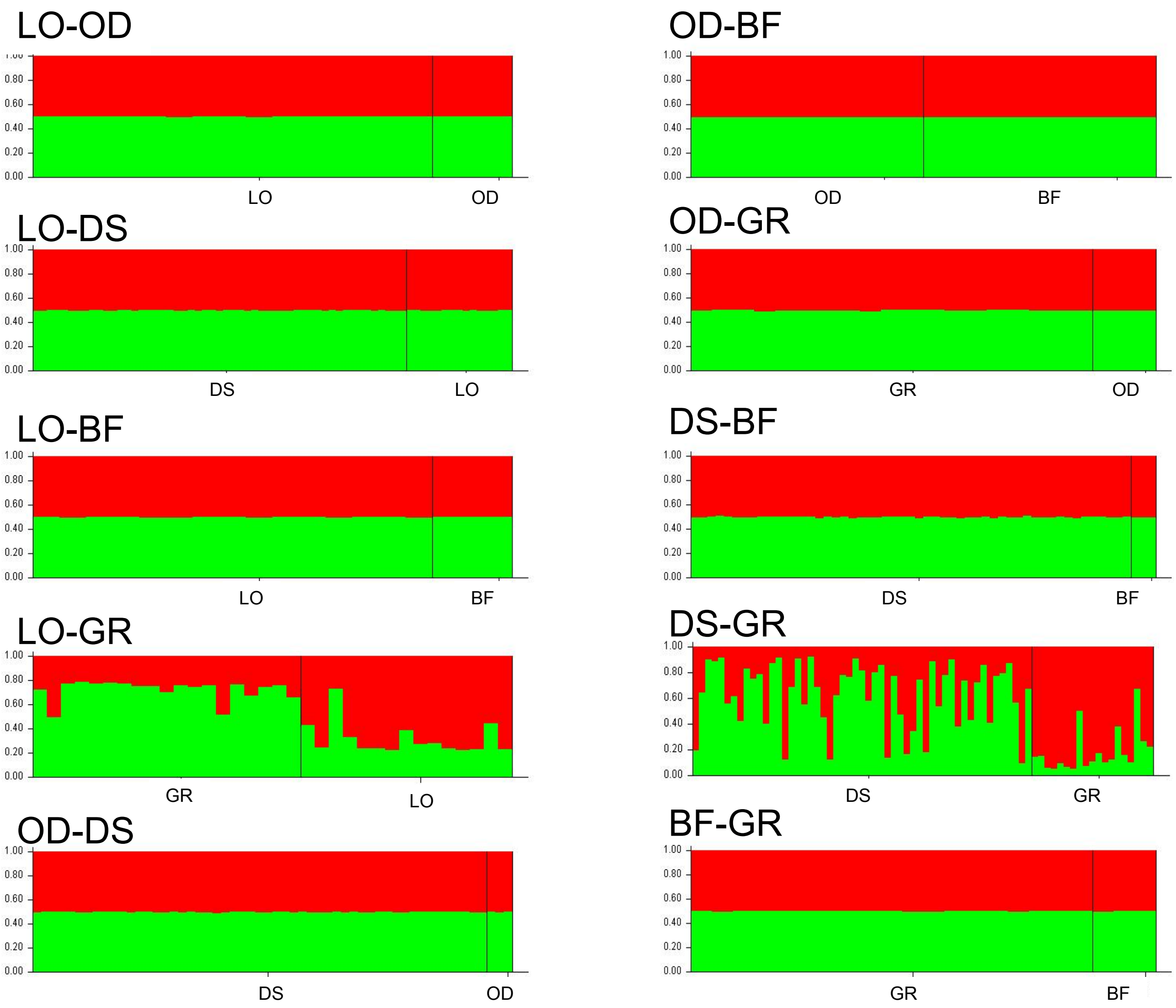
LO-OD
LO
OD
LO-DS
DS
LO
LO-BF
LO
BF
LO-GR
GR
LO
OD-DS
DS
OD
OD-BF
OD
BF
OD-GR
GR
OD
DS-BF
DS
BF
DS-GR
DS
GR
BF-GR
GR
BF
1.00
0.80
0.60
0.40
0.20
0.00

Figure S4. (A) Factorial correspondence analyses (FCA) of African forest and savanna elephants. Forest elephants were grouped by locality; savanna elephants were combined into a single group. Coordinate 1 explained 64.32% of the genetic variation and separated forest and savanna elephants (the latter highlighted in orange). Coordinate 2 explained 10.91% of the variance, and it separated forest elephant localities into western Congolian forest group (DS, OD, and LO, highlighted in darker green) and eastern Congolian forest group (BF and GR, highlighted with lighter green). Sierra Leone (SL) in West Africa was represented by a single individual. (B) Neighbor joining trees based on D_A (top), sample size bias corrected D_{ST} (middle), and sample size bias corrected F_{ST} (bottom) showed clear separation between forest elephants and savanna elephants (highlights are the same as in the previous panel). Western Congolian forest localities (DS, OD, and LO) and eastern Congolian forest localities (BF and GR) grouped separately, and a West African elephant from Sierra Leone (SL) was separated by a longer branch as well (also in Figure 3B). All bootstrap values higher than 70 are shown. Five forest-savanna elephant hybrids from GR were excluded from these analyses.

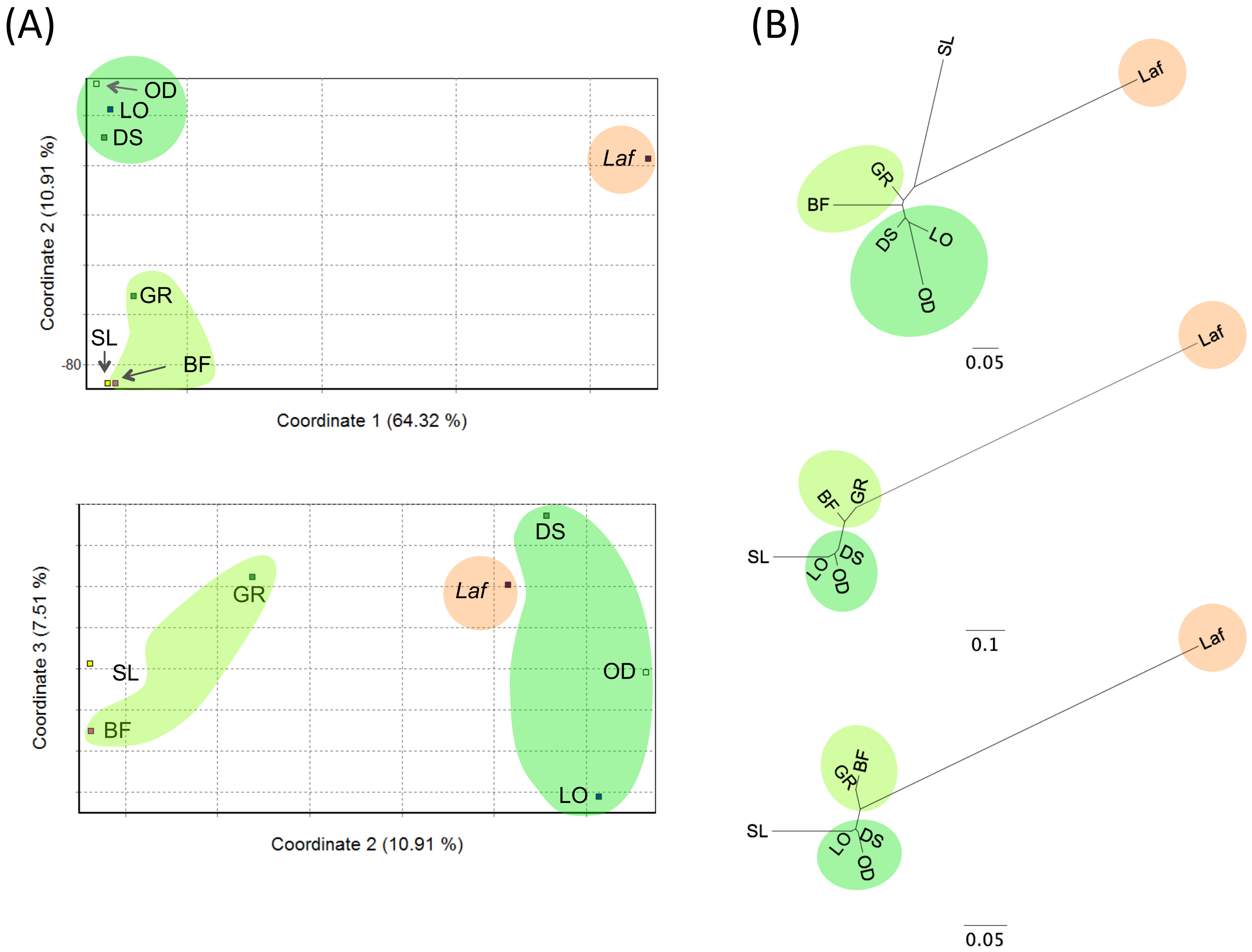
(A)
OD
LO
DS
Laf
GR
SL
BF
-80
Coordinate 2 (10.91 %)
Coordinate 1 (64.32 %)
DS
GR
Laf
SL
OD
BF
LO
Coordinate 3 (7.51 %)
Coordinate 2 (10.91 %)
(B)
SL
Laf
GR
BF
DS
LO
OD
0.05
Laf
BF
GR
SL
DS
LO
OD
0.1
Laf
BF
GR
SL
LO
DS
OD
0.05

Figure S5. Principal coordinate analyses (PCoA) were conducted to examine the genetic relationships among elephant individuals. (A) PCoA revealed clear separation between savanna and forest elephants (percentage of variation explained by coordinate 1: 25.03; coordinate 2: 5.28; coordinate 3: 4.15), with hybrid elephant GR0021 labeled. (B) PCoA based only on individual forest elephants, without savanna elephants, did not reveal clear subdivisions among forest elephants from different localities (coordinate 1: 25.16; coordinate 2: 9.18; coordinate 3: 16.38). Five forest-savanna hybrid elephants from Garamba were included in the analyses involving both species, but not in the analyses involving only forest elephants.

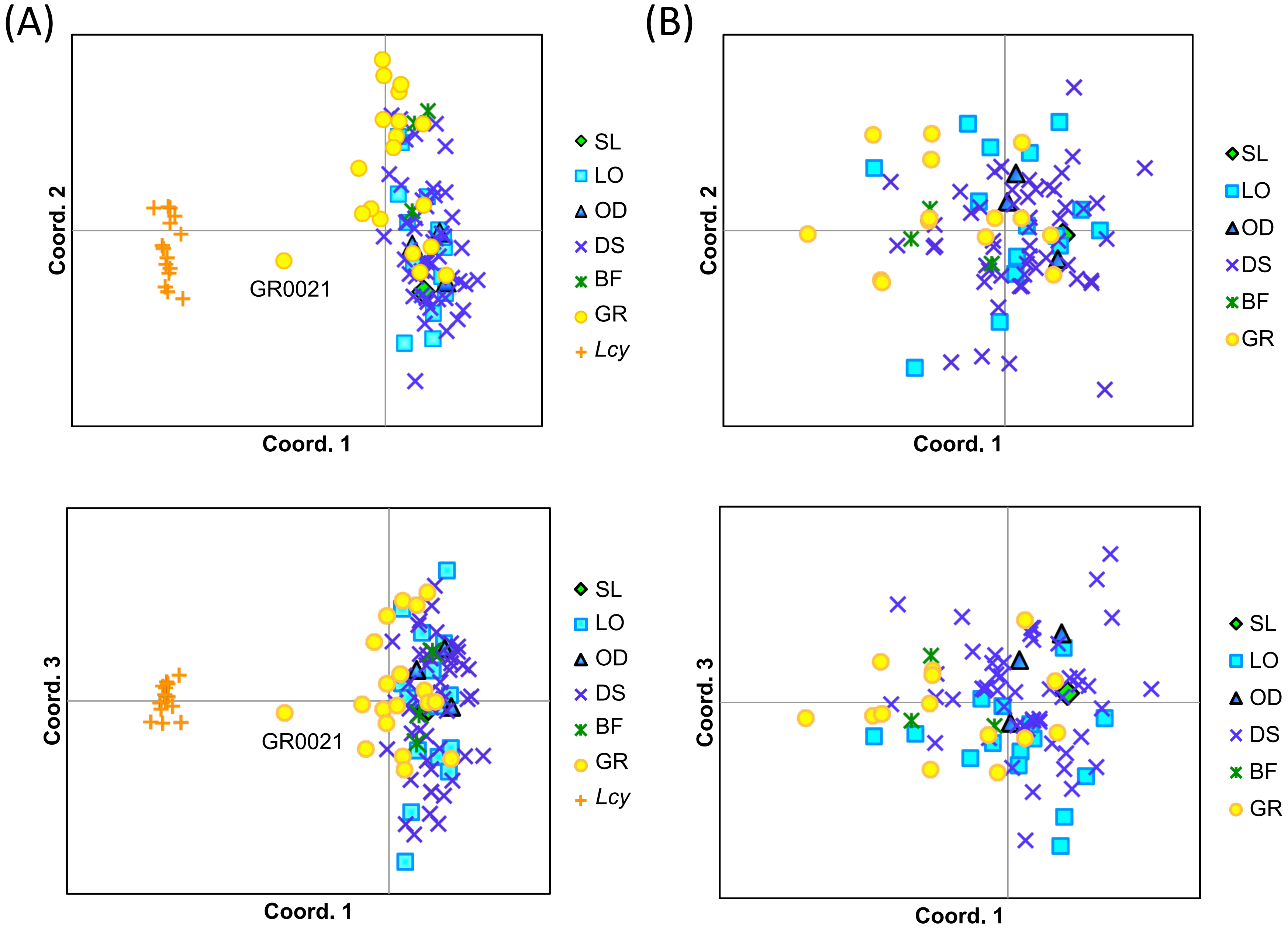

(A)
(B)
Coord. 1
Coord. 2
Coord. 3
GR0021
SL
LO
OD
DS
BF
GR
Lcy

Figure S6. Neighbor joining trees used to test the effect of using a sample size of one elephant from a locality: (A) including savanna elephants, and (B) using only forest elephant samples. Each tree was inferred using a single individual elephant from Lope National Park (LO) that was randomly chosen. Nine trees were inferred: a different LO elephant was randomly chosen three different times. For each individual, a tree was inferred based on three different genetic distance calculation methods: in the top panel, D_A; in the middle panel, sample size bias corrected D_{ST}; and in the bottom panel, sample size bias corrected F_{ST}. Different colors were used to label the single individual from Sierra Leone (SL) and the individual chosen from LO. The sample size of one led to a longer branch length for the LO individual when the genetic distance D_A was used. The branch length was not longer for the LO individual in the trees relying on methods that account for sample-size bias: D_{ST} and F_{ST}. By contrast, the single Sierra Leone individual, the only one in our sample set from the West African Guinean forest block, was placed at the end of a long branch consistently compared to other localities, regardless of sample size correction.

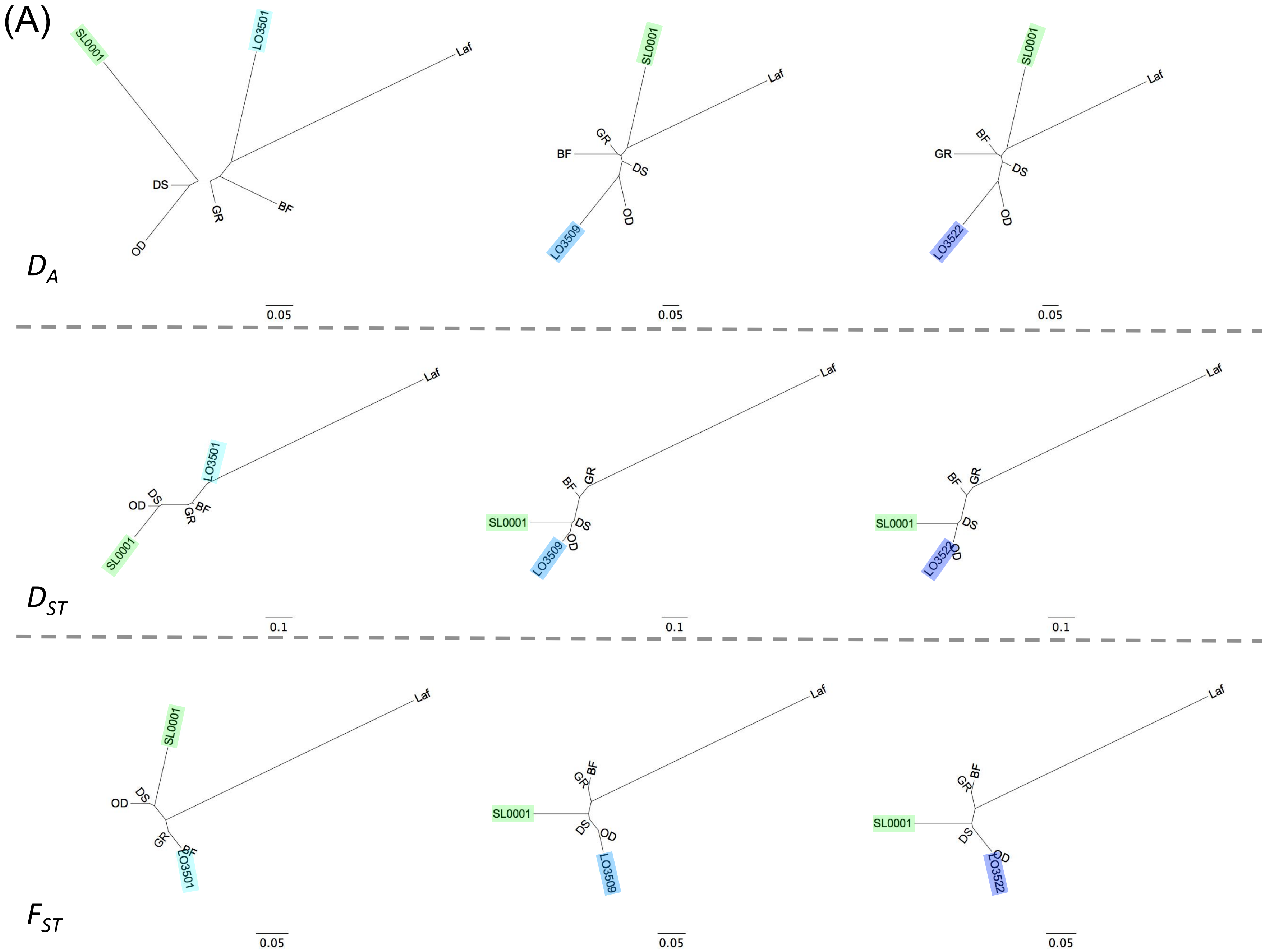
(A)
SL0001
LO3501
Laf
DS
GR
BF
OD
LO3509
LO3522
0.05
0.1
D_A
D_{ST}
F_{ST}

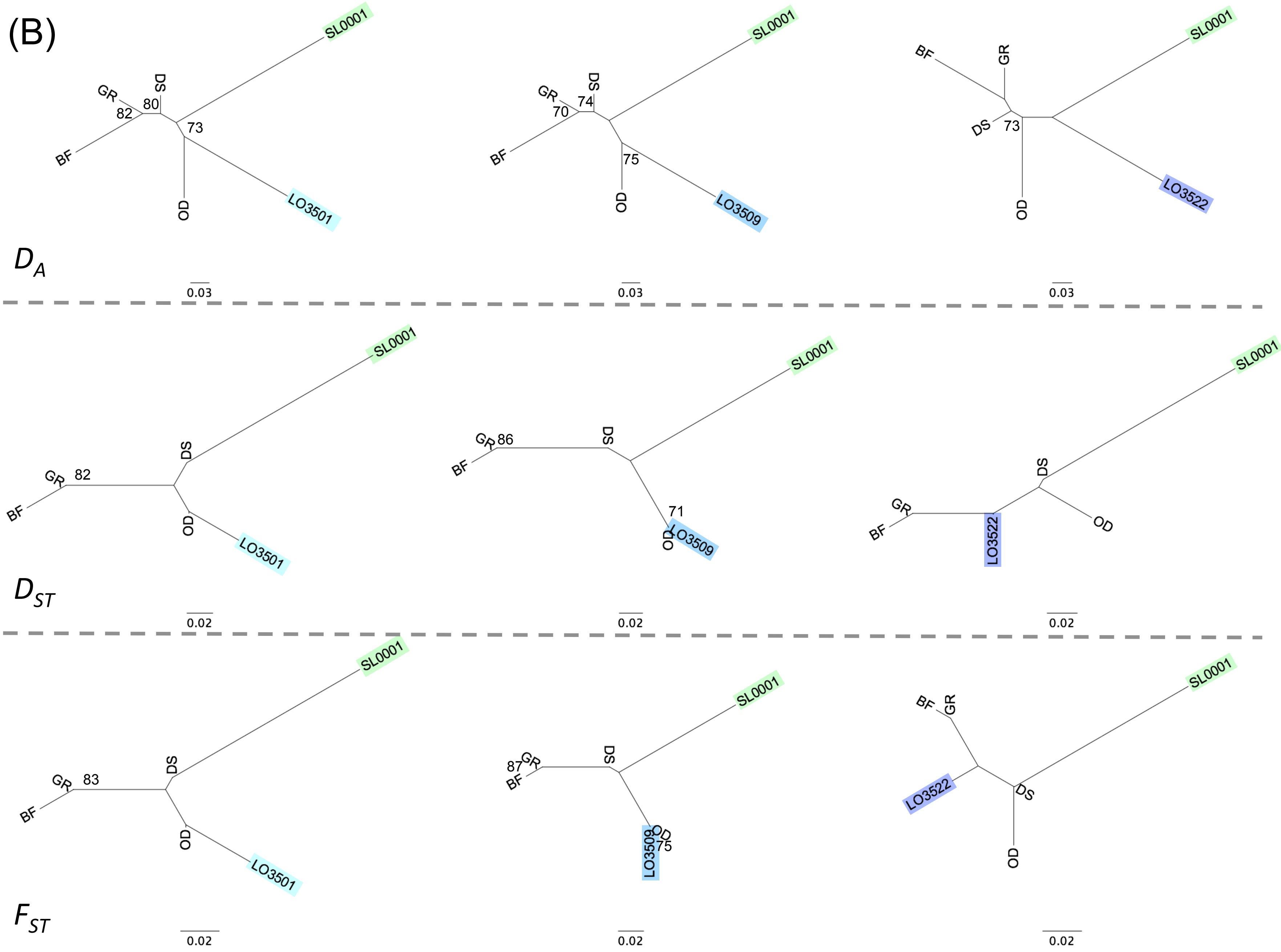

(B)
SL0001
GR
DS
80
82
73
BF
OD
LO3501
0.03
SL0001
GR
DS
70
74
BF
75
OD
LO3509
0.03
BF
GR
SL0001
DS
73
OD
LO3522
0.03
D_A
SL0001
DS
GR
82
BF
OD
LO3501
0.02
SL0001
GR
86
DS
BF
71
OD
LO3509
0.02
SL0001
DS
GR
BF
LO3522
OD
0.02
D_{ST}
SL0001
DS
GR
83
BF
OD
LO3501
0.02
SL0001
87
GR
DS
BF
LO3509
OD
75
0.02
BF
GR
SL0001
LO3522
DS
OD
0.02
F_{ST}

Figure S7. The relationships between genetic distances and geographic distances were examined for forest elephants from six geographic localities. Forest-savanna hybrid elephants from Garamba were excluded from these analyses. These results include the single West African Guinean forest block sample from Sierra Leone; similar analyses excluding this sample are shown in Figure 4. (A) Spatial autocorrelation analysis showed greater genetic similarity among forest elephants that were geographically close. (B) Marginally significant correlation between genetic distance (F_{ST}) and geographic distance was detected by comparing each pair of localities. Elephant localities separated by greater geographic distances had greater genetic distances ($r = 0.45$, $p = 0.049$). (C) Marginally significant correlation was also detected between genetic distance and geographic distance based on Rousset's distance $F_{ST}/(1\text{-}F_{ST})$ ($r = 0.45$, $p = 0.052$).

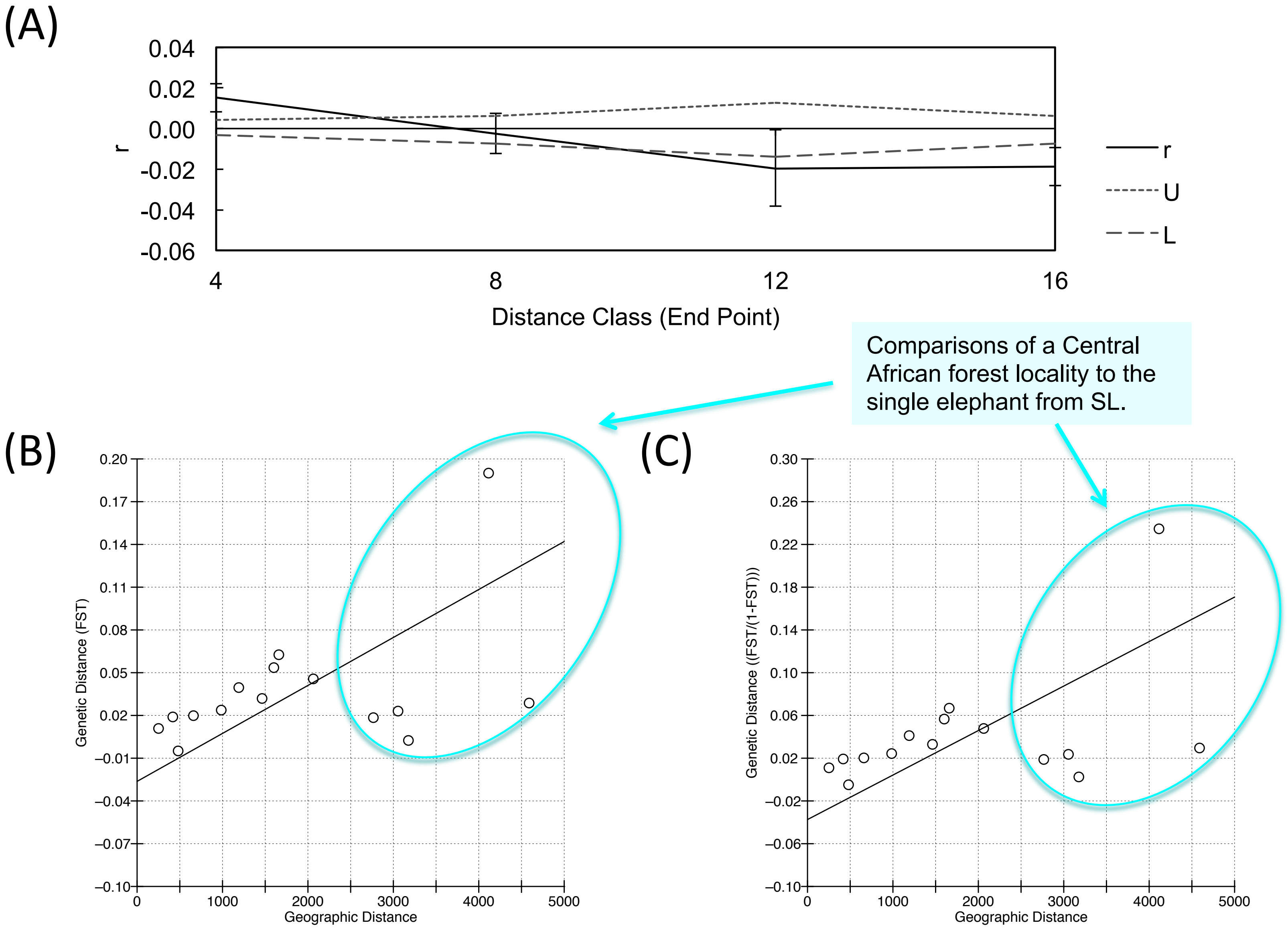

(A)
0.04
0.02
0.00
-0.02
-0.04
-0.06
r
4
8
12
16
Distance Class (End Point)
r
U
L
Comparisons of a Central African forest locality to the single elephant from SL.
(B)
0.20
0.17
0.14
0.11
0.08
0.05
0.02
−0.01
−0.04
−0.07
−0.10
Genetic Distance (FST)
0
1000
2000
3000
4000
5000
Geographic Distance
(C)
0.30
0.26
0.22
0.18
0.14
0.10
0.06
0.02
−0.02
−0.06
−0.10
Genetic Distance ((FST/(1-FST)))
Geographic Distance

Figure S6

Figure S8. Map showing the geographic distribution of elephant mtDNA subclades across Africa, as previously reported (Ishida *et al.* 2013). African elephant samples were grouped geographically into the localities represented by the large pie charts, which show the frequency at each of the localities of the eight major mtDNA subclades identified (Ishida *et al.* 2013). The small circles represent subclades present at locations sampled by previous mtDNA studies (Barriel *et al.* 1999; Debruyne 2005; Debruyne *et al.* 2003; Eggert *et al.* 2002; Johnson *et al.* 2007; Nyakaana *et al.* 2002), which were assigned using diagnostic sites for subclades, and confirmed as clustering together by Ishida and colleagues (Ishida *et al.* 2013). The inset map shows tropical forest (dark green) and mixed (light green) habitat zones (White 1983), which correspond approximately to the range of the forest elephant. Sampling locations in tropical forest habitats were as follows: DS, Dzanga Sangha, Central African Republic; OD, Odzala, Republic of Congo; BF, Bili Forest, Democratic Republic of Congo; LO, Lope, Gabon; and SL, Sierra Leone (one zoo individual). Savanna locations: CH, Chobe, MA, Mashatu; SA-Savuti in Botswana; BE, Benoue; WA, Waza in Cameroon; AB, Aberdares; AM, Amboseli; KE, Central Kenya/Laikipia; MK, Mount Kenya in Kenya; NA, Northern Namibia/Etosha; KR, Kruger in South Africa; NG, Ngorongoro; SE, Serengeti; TA, Tarangire in Tanzania; and HW, Hwange; SW, Sengwa, ZZ, Zambezi in Zimbabwe. GR, Garamba is located in the Guinea-Congolian/Sudanian transition zone of vegetation in D.R. Congo that historically included a mixture of forest and secondary grasslands (White 1983) suitable for both African elephant groups (Groves & Grubb 2000). This figure was published previously by Ishida and colleagues (Ishida *et al.* 2013); we copy it here without changes for information purposes, as permitted under the terms of the Creative Commons Attribution License (https://creativecommons.org/licenses/by-nc/3.0/legalcode).

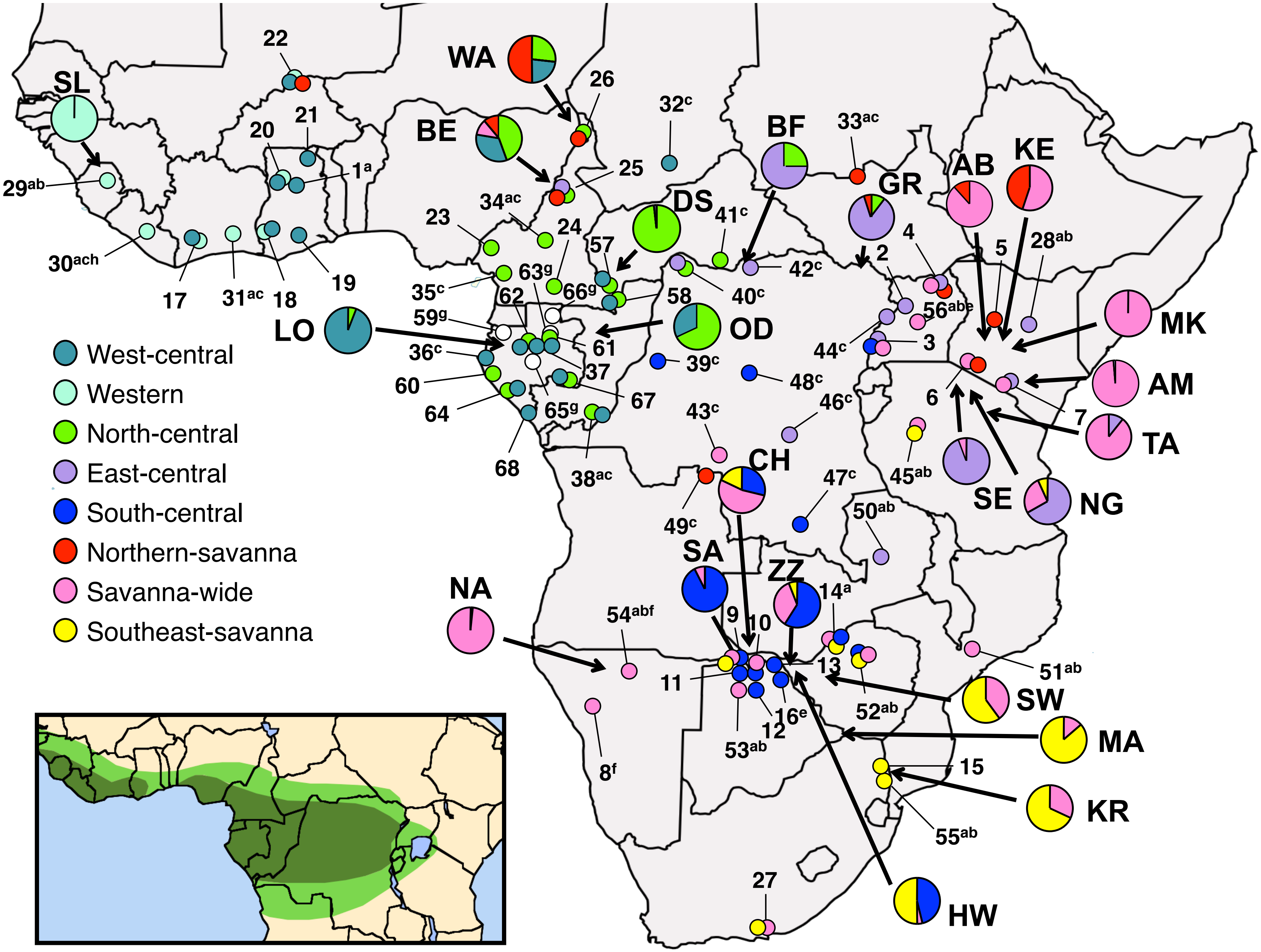

West-central
Western
North-central
East-central
South-central
Northern-savanna
Savanna-wide
Southeast-savanna
SL
WA
BE
BF
DS
GR
AB
KE
LO
OD
MK
AM
TA
SE
NG
CH
SA
ZZ
NA
SW
MA
KR
HW

Figure S9. Ecoregions within and near the Congolian forest block. The Congo Basin has been divided into five ecoregions of humid forest: Atlantic equatorial coastal forest, northwest (NW) Congolian lowland forests, Congolian swamp forests, central Congolian lowland forests, and northeastern (NE) Congolian lowland forests. There are three additional ecoregions of forest-savanna mosaic in the surrounding region: northern Congolian forest-savanna mosaic, western Congolian forest-savanna mosaic, and southern Congolian forest-savanna mosaic. The map was based on the Global Forest Atlas (http://globalforestatlas.yale.edu/congo/ecoregions/congo-basin-ecoregion) and on (Olson *et al.* 2001). We also indicate the approximate locations of the Cameroonian highlands forests and the Albertine rift montane forests.

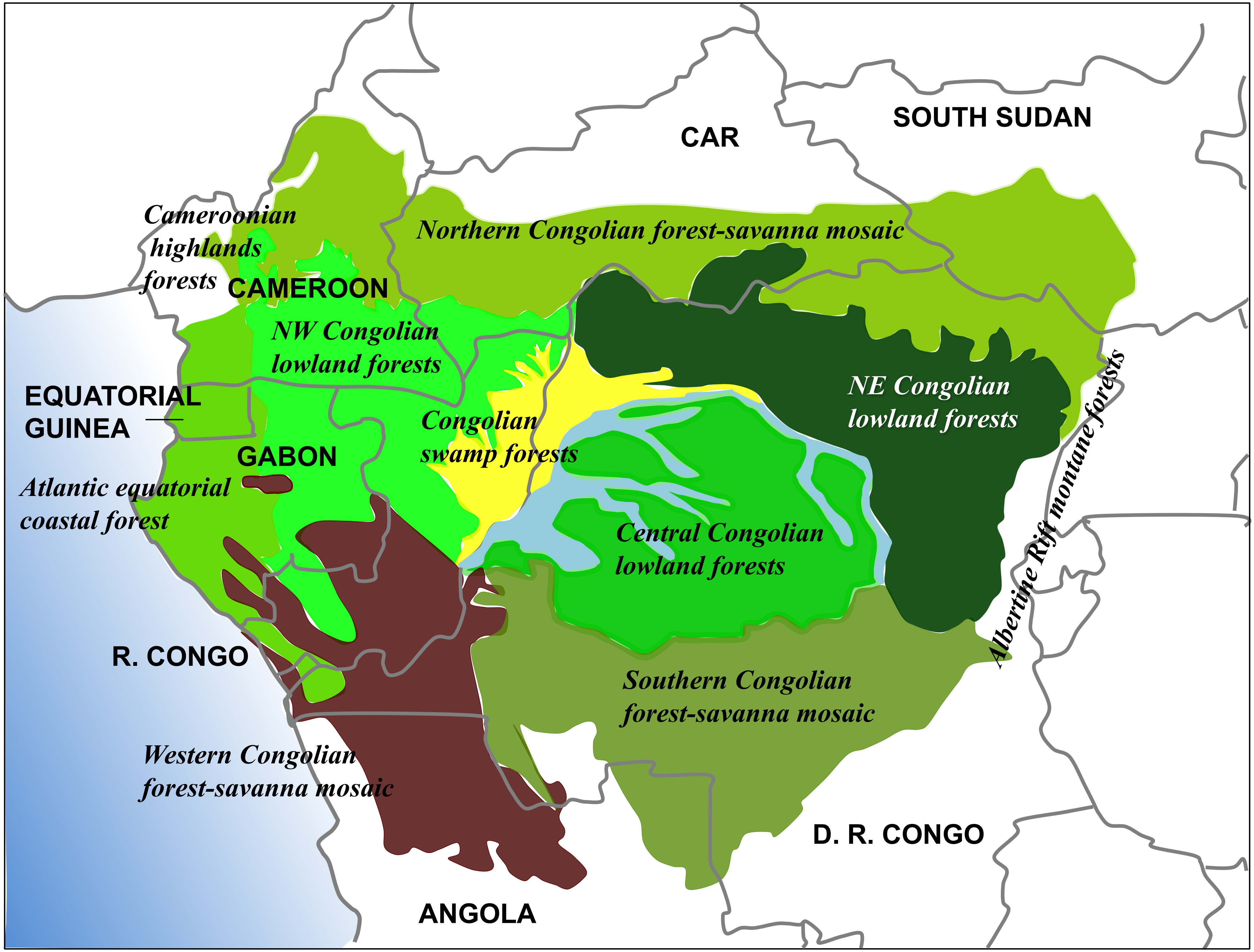

SOUTH SUDAN
CAR
Cameroonian highlands forests
CAMEROON
Northern Congolian forest-savanna mosaic
NW Congolian lowland forests
EQUATORIAL GUINEA
NE Congolian lowland forests
Congolian swamp forests
GABON
Atlantic equatorial coastal forest
Central Congolian lowland forests
Albertine Rift montane forests
R. CONGO
Southern Congolian forest-savanna mosaic
Western Congolian forest-savanna mosaic
D. R. CONGO
ANGOLA

Figure S10. Reproduction of a map showing the endemism richness of plants in Africa based on the distributions of 5881 species. The endemism richness is the sum of species in each grid, but with every species given weight in inverse proportion to the size of its geographic distribution, as described by (Linder 2014). Purple represents the highest value of endemism (many locally distributed species) while yellow represents the lowest value. This map was published previously by Linder and colleagues (Linder 2014); we copy it here unchanged for information purposes, as permitted under the terms of the Creative Commons Attribution License (https://creativecommons.org/licenses/by/3.0/).

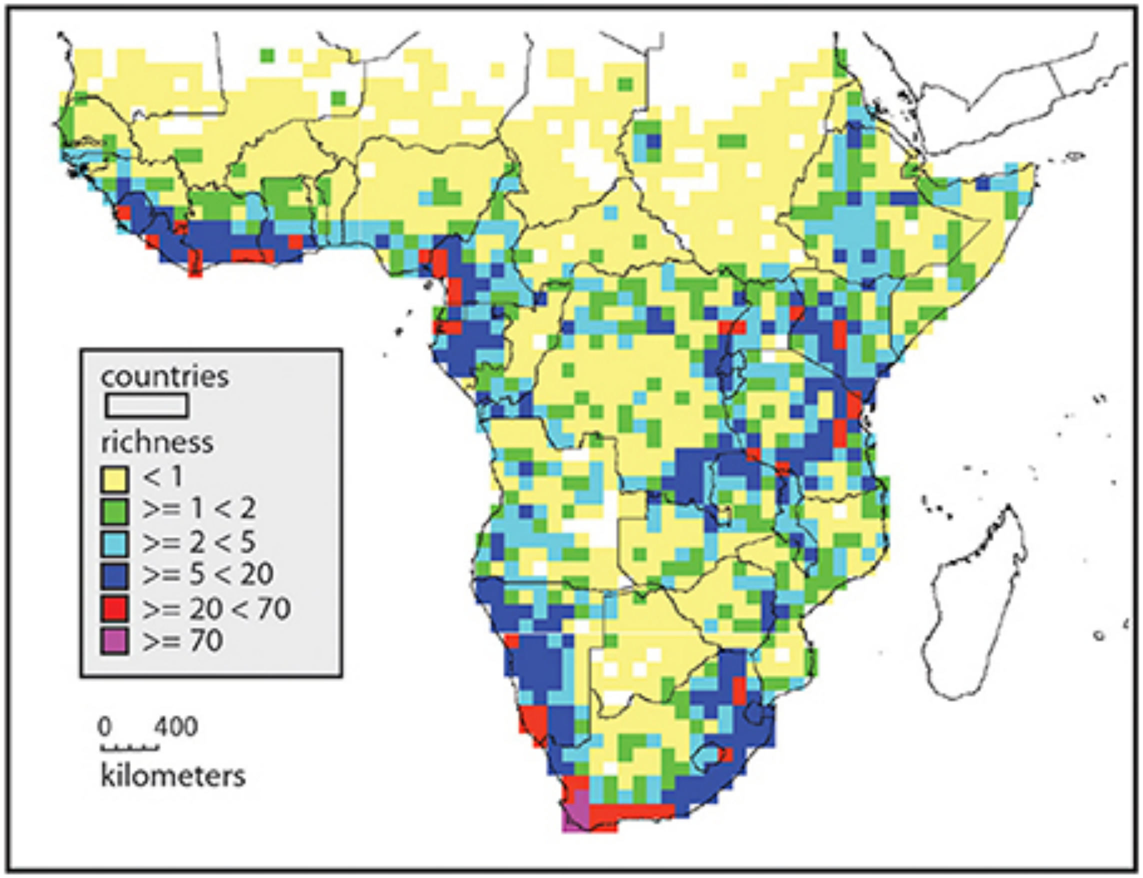
countries
richness
< 1
>= 1 < 2
>= 2 < 5
>= 5 < 20
>= 20 < 70
>= 70
0 400
kilometers

Supplementary information references

Barriel V, Thuet E, Tassy P (1999) Molecular phylogeny of Elephantidae. Extreme divergence of the extant forest African elephant. *C R Acad Sci III* **322**, 447-454.

Debruyne R (2005) A case study of apparent conflict between molecular phylogenies: the interrelationships of African elephants. *Cladistics* **21**, 31-50.

Debruyne R, Barriel V, Tassy P (2003) Mitochondrial cytochrome b of the Lyakhov mammoth (Proboscidea, Mammalia): new data and phylogenetic analyses of Elephantidae. *Mol Phylogenet Evol* **26**, 421-434.

Earl D, vonHoldt B (2012) STRUCTURE HARVESTER: a website and program for visualizing STRUCTURE output and implementing the Evanno method. *Conservation Genetics Resources* **4**, 359-361.

Eggert LS, Rasner CA, Woodruff DS (2002) The evolution and phylogeography of the African elephant inferred from mitochondrial DNA sequence and nuclear microsatellite markers. *Proc R Soc Lond B Biol Sci* **269**, 1993-2006.

Groves CP, Grubb P (2000) Do *Loxodonta cyclotis* and *L. africana* interbreed? *Elephant* **2**, 4-7.

Ishida Y, Georgiadis NJ, Hondo T, Roca AL (2013) Triangulating the provenance of African elephants using mitochondrial DNA. *Evol Appl* **6**, 253-265.

Johnson MB, Clifford SL, Goossens B*, et al.* (2007) Complex phylogeographic history of central African forest elephants and its implications for taxonomy. *BMC Evol Biol* **7**, 244.

Linder HP (2014) The evolution of African plant diversity. *Frontiers in Ecology and Evolution* **2**, 38.

Nyakaana S, Arctander P, Siegismund HR (2002) Population structure of the African savannah elephant inferred from mitochondrial control region sequences and nuclear microsatellite loci. *Heredity* **89**, 90-98.

Olson DM, Dinerstein E, Wikramanayake ED*, et al.* (2001) Terrestrial ecoregions of the world: a new map of life on earth: a new global map of terrestrial ecoregions provides an innovative tool for conserving biodiversity. *BioScience* **51**, 933-938.

Pritchard JK, Stephens M, Donnelly P (2000) Inference of population structure using multilocus genotype data. *Genetics* **155**, 945-959.

White F (1983) *The Vegetation of Africa* UNESCO, Paris.
